# Study on the Therapeutic Effects of Ginsenoside Rh2 in Asthma Improvement

**DOI:** 10.3390/ijms27188160

**Published:** 2026-09-14

**Authors:** Yuming Wang, Xiaohua Li, Dongming Xue, Tianxia Sun, Yu Zhao

**Affiliations:** Jilin Ginseng Academy, Changchun University of Chinese Medicine, Changchun 130117, China; 18940393301@163.com (Y.W.); xiaohua130319@163.com (X.L.); xuedongming0119@163.com (D.X.)

**Keywords:** Ginsenoside Rh2, asthma, lipid metabolism, inflammation

## Abstract

Ginsenoside Rh2, a protopanaxadiol-type saponin derived from Panax ginseng, exhibits a broad spectrum of biological activities; however, its specific mechanisms in asthma remain elusive. In this study, we systematically evaluated the therapeutic efficacy of Rh2 against asthma using both in vitro and in vivo models. A lipopolysaccharide (LPS)-induced in vitro cell model was established and treated with low, medium, and high doses of Rh2 (1, 5, and 10 μg/mL). Concurrently, an in vivo asthma model was constructed via ovalbumin (OVA) sensitization and administered with three doses of Rh2 (2.5, 5, and 10 mg/kg). The results demonstrated that Rh2 significantly suppressed pro-inflammatory cytokines (interleukin-4 [IL-4], interleukin-5 [IL-5], interleukin-13 [IL-13], interleukin-33 [IL-33], and tumor necrosis factor-α [TNF-α]) and upregulated anti-inflammatory factors (interferon-γ [IFN-γ] and interleukin-10 [IL-10]) in both experimental models. Furthermore, Rh2 inhibited the expression of key inflammatory pathway proteins, including suppression of tumorigenicity 2 (ST2), Toll-like receptor 4 (TLR4), myeloid differentiation primary response 88 (MyD88), nuclear factor kappa-light-chain-enhancer of activated B cells (NF-κB), and c-Jun N-terminal kinase (JNK). Transcriptomic and metabolomic analyses revealed that Rh2 primarily modulates the TNF-α signaling pathway, cytokine–cytokine receptor interaction, pyrimidine metabolism, and glycine, serine, and threonine metabolism. Integrated analysis further identified significant correlations between differentially expressed genes (*C-X-C motif chemokine ligand 2* [*CXCL2*], *C-X-C motif chemokine ligand 17* [*CXCL17*], and *leukemia inhibitory factor* [*LIF*]) and differential metabolites (cytosine, L-histidine, and creatine). Collectively, these findings suggest that ginsenoside Rh2 ameliorates asthma by modulating cytokine interactions and amino acid metabolism.

## 1. Introduction

Asthma remains one of the most common chronic respiratory disorders worldwide and continues to pose a substantial global health burden [1]. It is projected that the prevalence of asthma will remain persistently high globally over the coming decades [2]. The pathogenesis of asthma primarily involves the interaction between chronic airway inflammation and airway hyperresponsiveness, both of which are largely driven by type 2 immune responses [3]. This inflammatory response is mediated by Th2 cells and type 2 innate lymphocytes (ILC2s), which secrete cytokines such as IL-4, IL-5, and IL-13, leading to pathological changes including eosinophil infiltration, Immunoglobulin E (IgE) production, excessive mucus secretion, and airway remodeling.

Currently, asthma medications are primarily categorized into control agents and relievers. Control agents, predominantly inhaled corticosteroids (ICS), represent highly effective anti-inflammatory drugs in contemporary asthma management [4]. However, while ICS effectively suppress airway inflammation to control symptoms, they do not provide a definitive reverse the underlying pathological processes of asthma, and symptom exacerbation may occur upon discontinuation. Relievers function by rapidly alleviating bronchospasm and exercise-induced bronchoconstriction, making them essential during acute asthma exacerbations. Nevertheless, these medications should be used intermittently at the lowest effective dose, as excessive use may induce adverse effects such as tachycardia or tremors [5]. Thus, although numerous asthma medications are available today, none offer a complete cure, and all carry potential side effects.

Traditional Chinese medicine (TCM) exerts multi-target regulatory effects on immunity, along with anti-inflammatory and anti-asthmatic actions. It improves airway hyperresponsiveness and demonstrates relatively lower rates of adverse reactions. Certain TCM components, such as ephedra alkaloids and glycyrrhizin, exhibit hormone-like effects but with milder actions, suggesting their potential suitability for long-term regulation [6]. Recent studies have revealed that specific TCM formulations can modulate the T helper 1/T helper 2 (Th1/Th2) immune balance and reduce the release of inflammatory mediators, thereby alleviating asthmatic responses [7]. Although conventional TCM preparations exhibit a slow onset of action and were previously unsuitable for acute asthma episodes, advances in extraction technologies and drug delivery systems have continuously improved TCM formulations, providing novel approaches for personalized asthma treatment.

*Panax ginseng* is a perennial herbaceous plant belonging to the Araliaceae family and the *Panax genus*. It is a precious medicinal material widely used in TCM, renowned for its traditional therapeutic effects such as replenishing primordial qi, restoring pulse and stabilizing collapse, tonifying the spleen and lungs, promoting fluid and blood production, and calming the mind and enhancing intelligence [8]. Modern pharmacological investigations have attributed its biological activities primarily to diverse active constituents, such as ginsenosides, polysaccharides, and amino acids, which exhibit effects including enhancing immune function, improving cardiovascular function, regulating the central nervous system, and anti-fatigue [9,10,11]. Research has demonstrated that certain ginsenosides significantly alleviate pulmonary inflammation. These compounds reduce the production of pro-inflammatory cytokines and mitigate oxidative stress by inhibiting relevant signaling pathways, and they also improve the intestinal immune environment, thereby reversing inflammatory states [12].

Ginsenoside Rh2, a representative rare ginsenoside, has attracted considerable interest because of its diverse pharmacological activities and potential therapeutic value in inflammatory pulmonary inflammation. In terms of immunomodulation, Rh2 influences key signaling pathways such as NF-κB, Mitogen-activated protein kinase (MAPK), and Signal transducer and activator of transcription 3 (STAT3). Its specific regulatory mechanisms have been clearly demonstrated across various disease models: for instance, in atopic dermatitis models, Rh2 effectively inhibits NF-κB-mediated Thymic stromal lymphopoietin (TSLP) expression while suppressing Th2 cell differentiation. These findings suggest that Rh2 possesses potential regulatory capabilities over the “epithelial-derived pyroptosis-type 2 inflammatory axis” [13]. In studies investigating myocardial ischemia or hypoxic injury, Rh2 effectively ameliorates the local inflammatory microenvironment and mitigates inflammatory damage to myocardial tissue by inhibiting NF-κB translocation, thereby suppressing the activation of NLR family pyrin domain-containing 3 (NLRP3) inflammasomes [14]. Additional research further demonstrates that Rh2 regulates pro-apoptotic molecules in cardiomyocytes (e.g., caspase-1 and Gasdermin D) via the Phosphoinositide 3-kinase/Protein kinase B (PI3K/AKT) pathway, highlighting its potential role in controlling inflammatory-cell death processes and expanding its clinical applications in inflammatory diseases [15]. These findings indicate that ginsenoside Rh2 modulates inflammatory responses through multiple pathways, providing a robust theoretical foundation for its therapeutic utilization in chronic inflammatory airway diseases such as asthma.

The novelty of this study lies in its comprehensive in vitro and in vivo evaluation of the therapeutic effects of Rh2. Furthermore, through integrated multi-omics analysis, we revealed that ginsenoside Rh2 ameliorates asthma by modulating inflammatory cytokines and amino acid metabolism. The goal is to facilitate the clinical translation of highly effective and low-toxicity natural active compounds, thereby providing potential therapeutic candidates for the prevention and treatment of respiratory diseases.

## 2. Results

### 2.1. Effect of Ginsenoside Rh2 on LPS Induced Cytokine Release in 16HBE Cells

**Figure 1:**
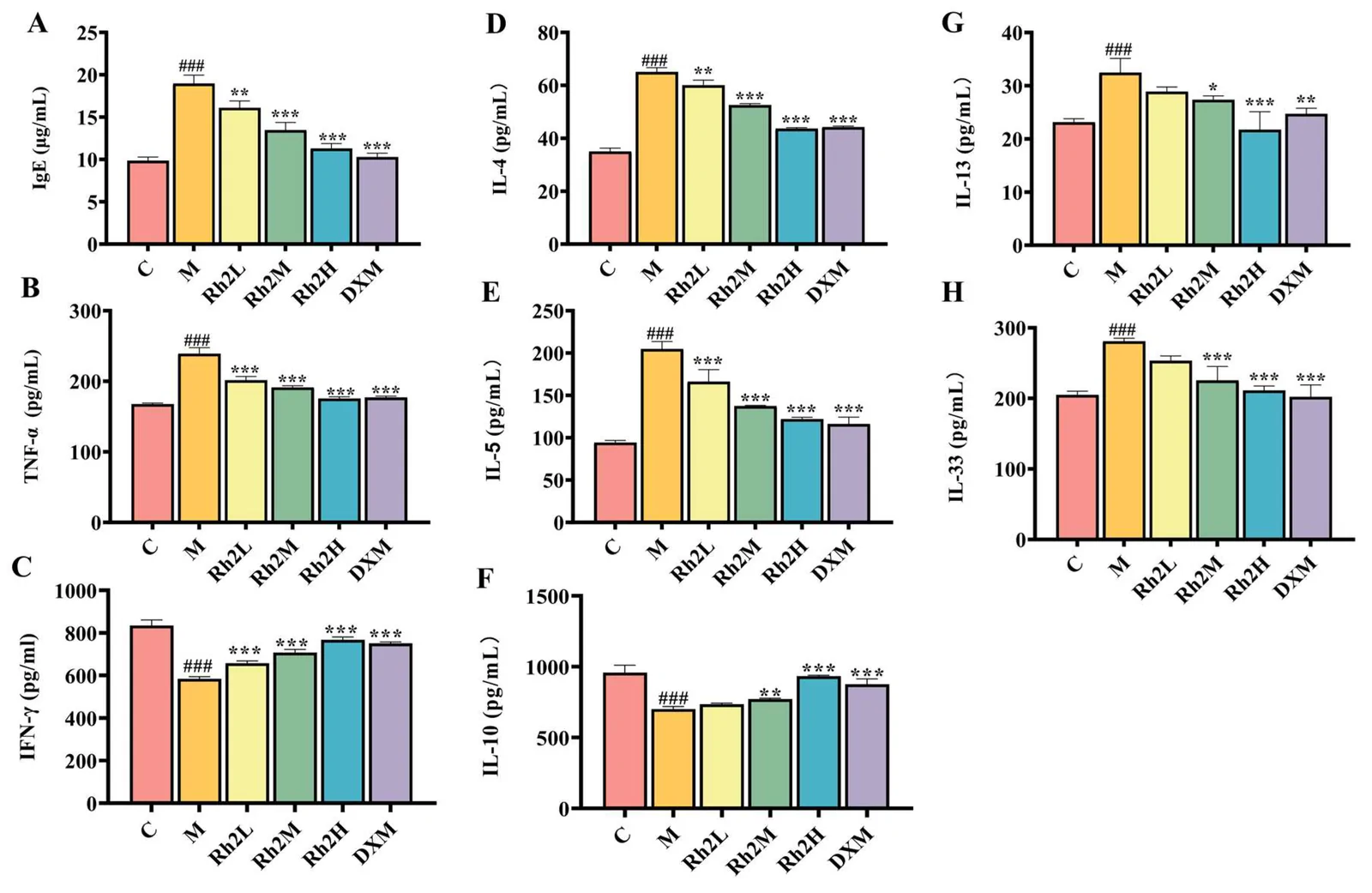
ELISA detection of cytokine levels (IgE, TNF-α, IL-4, IL-5, IL-13, IL-33, IFN-γ, and IL-10) in the supernatants of 16HBE cells from each group. Rh2 treatment reduced the levels of IgE (**A**), TNF-α (**B**), IL-4 (**D**), IL-5 (**E**), IL-13 (**G**), and IL-33 (**H**), while simultaneously increasing the levels of IFN-γ (**C**) and IL-10 (**F**). Data are presented as mean ± SD (*n* = 3). ### *p* < 0.001 vs. Group C; * *p* < 0.05, ** *p* < 0.01, *** *p* < 0.001 vs. Group M. Statistical analysis was performed using one-way ANOVA followed by Tukey’s post hoc test.

Cell viability was first evaluated to determine whether Rh2 exerted cytotoxic effects on 16HBE cells (Supplementary Figure S1). The levels of inflammatory mediators in the supernatants of all groups were measured using ELISA (Figure 1). Compared with the control group, the model group showed significantly increased secretion of pro-inflammatory factors IgE, TNF-α, IL-4, IL-5, IL-13, and IL-33 (### *p* < 0.001), whereas IFN-γ and IL-10 levels were significantly decreased (### *p* < 0.001). Compared with the model group, Rh2 treatment significantly reduced the levels of the pro-inflammatory mediators and restored the levels of IFN-γ and IL-10 (*** *p* < 0.001). These results indicate that Rh2 attenuated the inflammatory response in LPS-stimulated 16HBE cells.

### 2.2. Effect of Ginsenoside Rh2 on Oxidative Stress in 16HBE Cells

**Figure 2:**
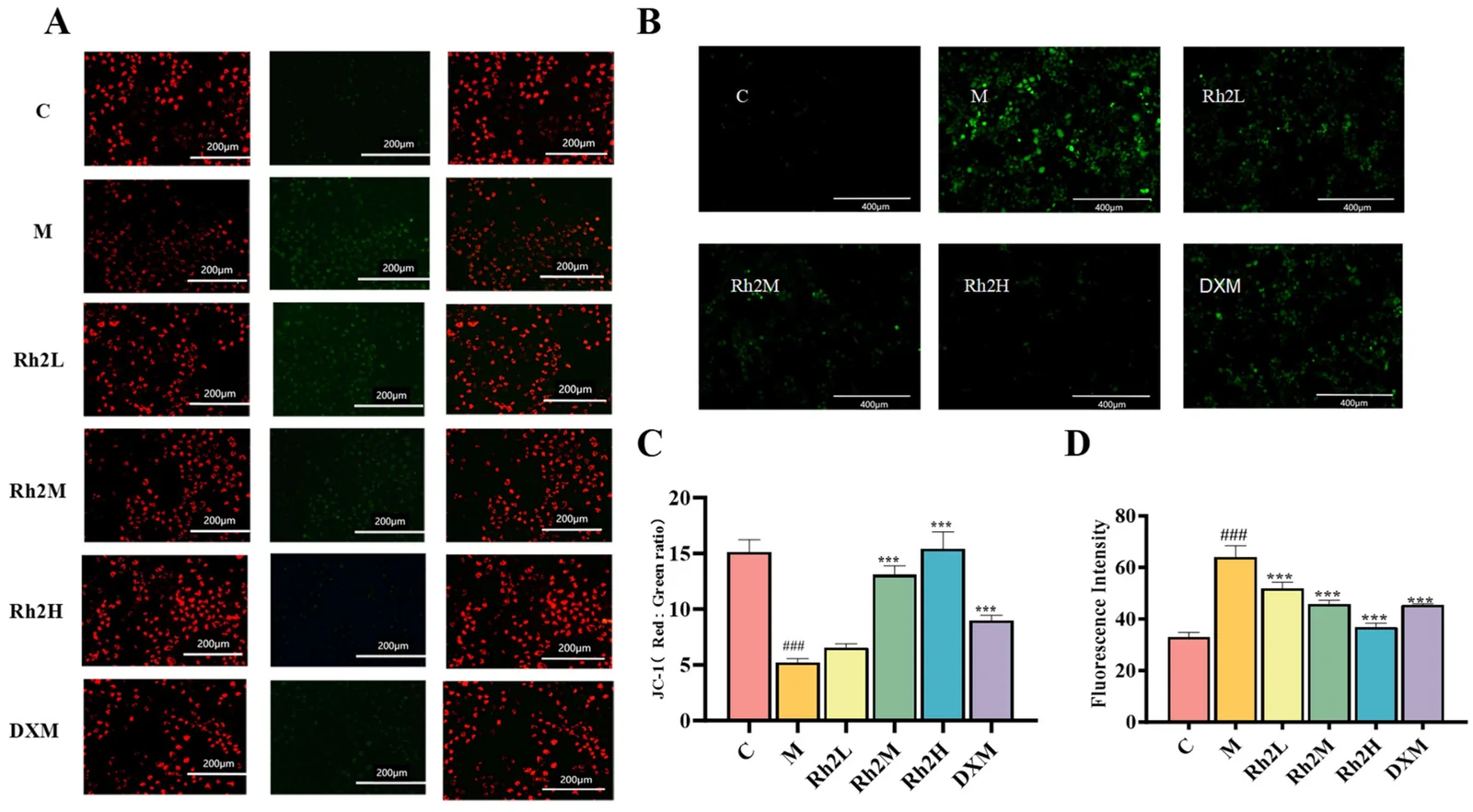
Effects of ginsenoside Rh2 on mitochondrial membrane potential and intracellular ROS levels in 16HBE cells. Mitochondrial membrane potential was assessed using the JC-1 fluorescent probe, and the red-to-green fluorescence ratio was quantified (**A**,**C**). Intracellular ROS levels were evaluated using the DCFH-DA probe (**B**,**D**). Data are presented as mean ± SD (*n* = 3). ### *p* < 0.001 vs. Group C; *** *p* < 0.001 vs. Group M. Statistical analysis was performed using one-way ANOVA followed by Tukey’s post hoc test.

Mitochondrial membrane potential was evaluated using the JC-1 assay (Figure 2A,C). Compared with the control group, the model group showed a significantly lower red-to-green fluorescence ratio than the control group (### *p* < 0.001), indicating mitochondrial membrane depolarization. Rh2 treatment significantly increased the red-to-green fluorescence ratio relative to the model group (*** *p* < 0.001), suggesting that Rh2 alleviated LPS-induced mitochondrial dysfunction.

Intracellular ROS levels were assessed using the DCFH-DA fluorescent probe (Figure 2B,D). ROS-associated green fluorescence was markedly increased in the model group compared with the control group (### *p* < 0.001). Rh2 treatment significantly reduced fluorescence intensity relative to the model group (*** *p* < 0.001), indicating attenuation of LPS-induced oxidative stress.

### 2.3. Regulatory Effects of Ginsenoside Rh2 on Inflammatory-Related Genes and Protein Expression in 16HBE Cells

**Figure 3:**
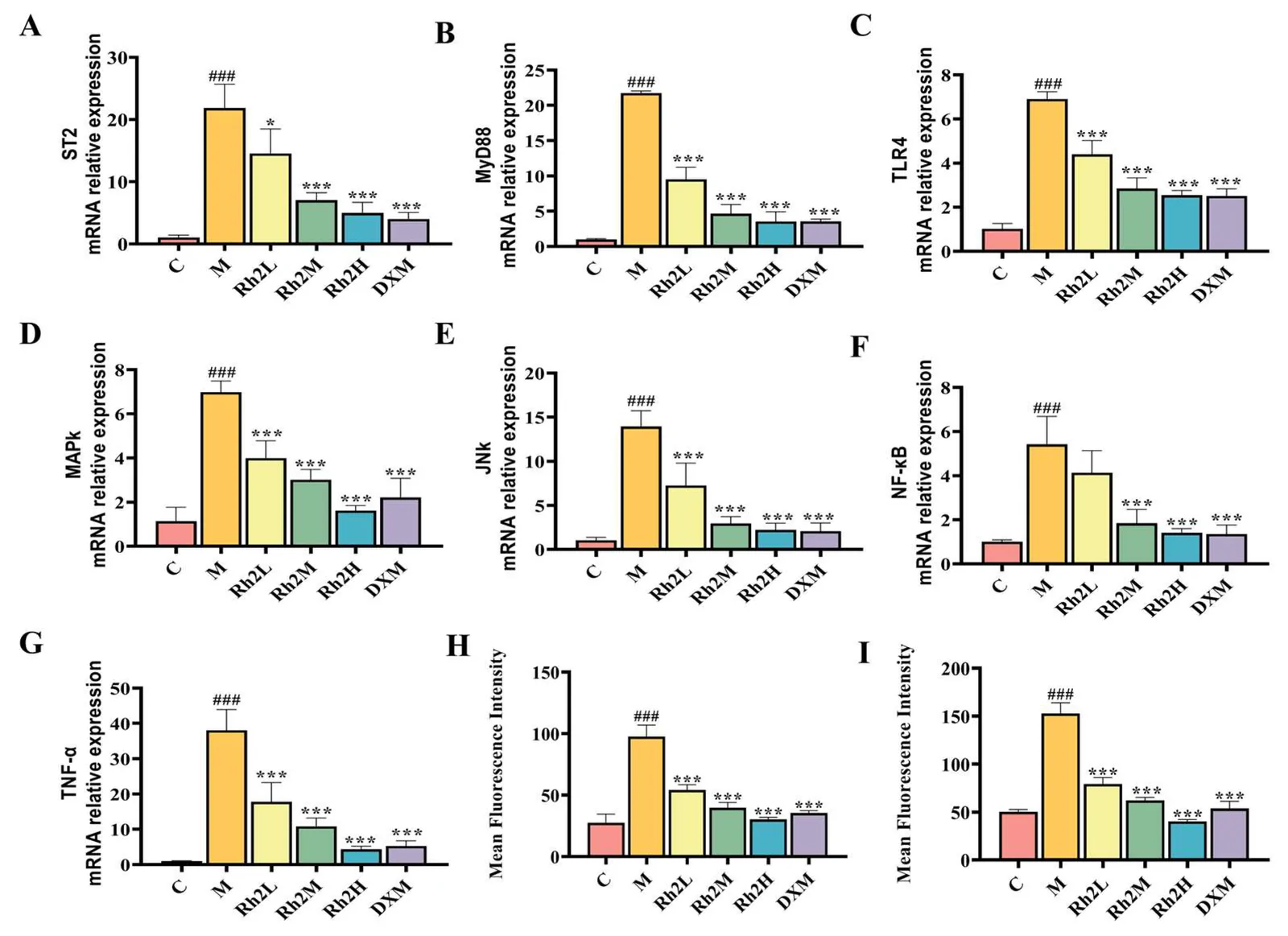

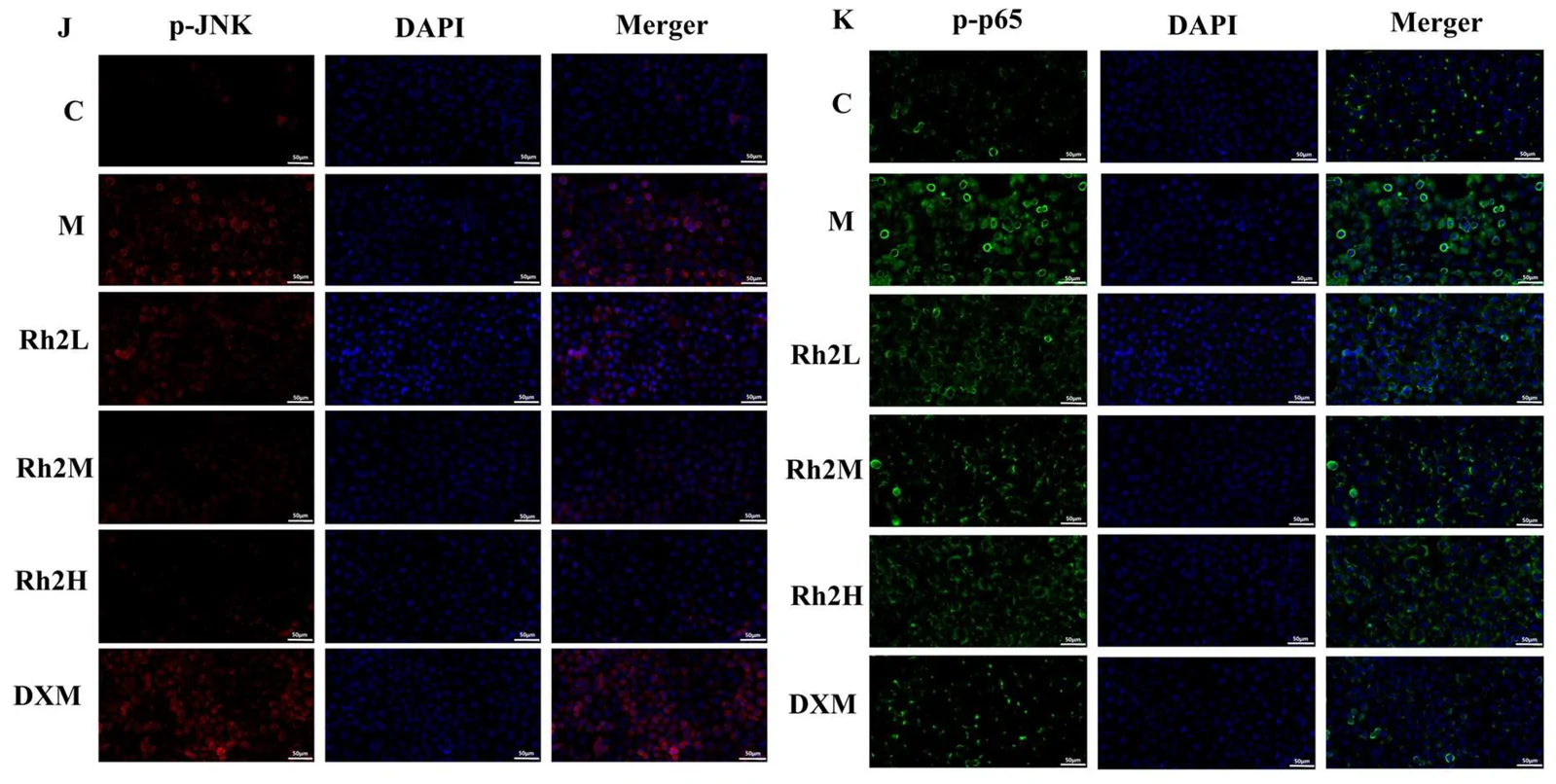
Effects of ginsenoside Rh2 on inflammatory signaling in 16HBE cells. The mRNA expression levels of *ST2*, *TLR4*, *MyD88*, *NF-κB*, *MAPK*, *JNK*, and *TNF-α* were determined by RT-qPCR (**A**–**G**). Immunofluorescence staining was used to evaluate the expression and intracellular localization of p-JNK and p-p65 (**H**–**K**). **Scale bars represent 50 µm. **Data are presented as mean ± SD (*n* = 3). ### *p* < 0.001 vs. Group C; * *p* < 0.05, *** *p* < 0.001 vs. Group M. One-way ANOVA followed by Tukey’s post hoc test was used for statistical analysis.

The mRNA expression of genes associated with inflammatory signaling was measured by reverse transcription quantitative real-time PCR (RT-qPCR) (Figure 3A–G). Compared with the control group, the model group exhibited significantly increased expression of ST2, TLR4, MyD88, NF-κB, MAPK, JNK, and TNF-α (### *p* < 0.001). Rh2 treatment significantly reduced the expression of these genes relative to the model group (*** *p* < 0.001).

To further assess the effect of ginsenoside Rh2 on inflammatory signal transduction, immunofluorescence staining was performed to detect phosphorylated c-Jun N-terminal kinase (p-JNK) and phosphorylated p65 (p-p65). In the model group, p-JNK fluorescence intensity was markedly increased and was predominantly localized in the nucleus (Figure 3H,J). Rh2 treatment reduced p-JNK fluorescence intensity. A similar pattern was observed for p-p65: the model group exhibited strong nuclear p-p65 fluorescence, whereas Rh2 treatment reduced both the fluorescence signal and its nuclear localization (Figure 3I,K). These findings suggest that Rh2 suppresses the activation and nuclear translocation of JNK- and NF-κB-related signaling components in LPS-stimulated 16HBE cells.

### 2.4. Effects of Ginsenoside Rh2 on Pulmonary Function and Pathological Damage in Mice

Following model establishment, model validity was preliminarily assessed by behavioral scoring during stimulation, body weight measurement, and organ coefficient analysis (Supplementary Tables S1 and S2 and Supplementary Figure S2). Airway hyperresponsiveness (AHR) was subsequently evaluated by acetylcholine challenge using a non-invasive whole-body plethysmography system. Compared with the control group, the model group exhibited a significantly increased respiratory rate (### *p* < 0.001), whereas Rh2 treatment significantly reduced the respiratory rate relative to the model group (*** *p* < 0.001). Rh2 treatment also significantly reduced AHR compared with the model group (*** *p* < 0.001). These findings indicate that Rh2 improved airway hyperresponsiveness in asthma model mice.

**Figure 4:**
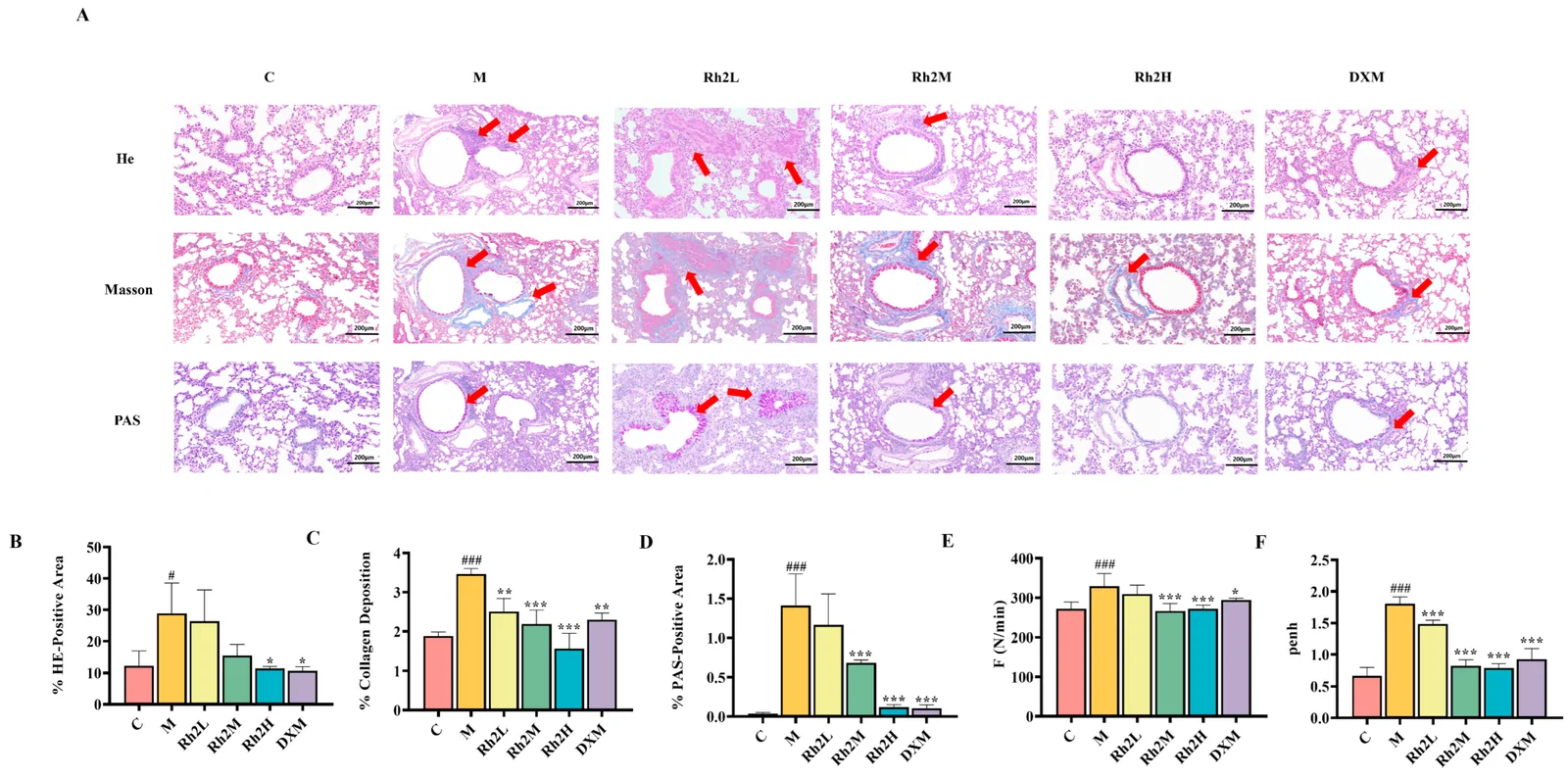
Assessment of lung function in mice using a live-scanning system. Rh2 treatment reduced respiratory rate (**B**) and airway hyperresponsiveness (**C**) in mice. Rh2 mitigated pathological changes in asthma model mice. HE staining (**A**,**D**) demonstrated that Rh2 decreased pulmonary inflammatory-cell infiltration. Masson staining (**A**,**E**) revealed reduced pulmonary collagen deposition. PAS staining (**A**,**F**) showed decreased goblet-cell hyperplasia and mucus secretion in the lungs. Values are mean ± SD, *n* = 3; one-way ANOVA, Tukey’s post hoc. Data are presented as mean ± SD (*n* = 3). # *p* < 0.001, ### *p* < 0.001 vs. Group C; * *p* < 0.05, ** *p* < 0.01, *** *p* < 0.001 vs. Group M. One-way ANOVA followed by Tukey’s post hoc test was used for statistical analysis.

Hematoxylin and eosin (H&E) staining showed that the airway epithelial structure was intact and that no obvious pathological alterations were present in the control group. In contrast, the model group exhibited marked inflammatory-cell infiltration and thickening of the airway basement membrane. Rh2 treatment markedly reduced inflammatory-cell infiltration (Figure 4A,D).

Masson’s trichrome staining showed significantly increased airway collagen deposition in the model group compared with the control group, indicating enhanced extracellular matrix deposition and airway remodeling. Rh2 treatment markedly reduced collagen deposition and alleviated pulmonary fibrotic changes (Figure 4A,E).

Periodic acid–Schiff (PAS) staining showed pronounced goblet-cell hyperplasia and excessive mucus secretion in the model group compared with the control group. Rh2 treatment markedly attenuated goblet-cell hyperplasia and mucus hypersecretion (Figure 4A,F).

### 2.5. Effect of Ginsenoside Rh2 on the Number of Inflammatory Cells In Vivo

**Figure 5:**
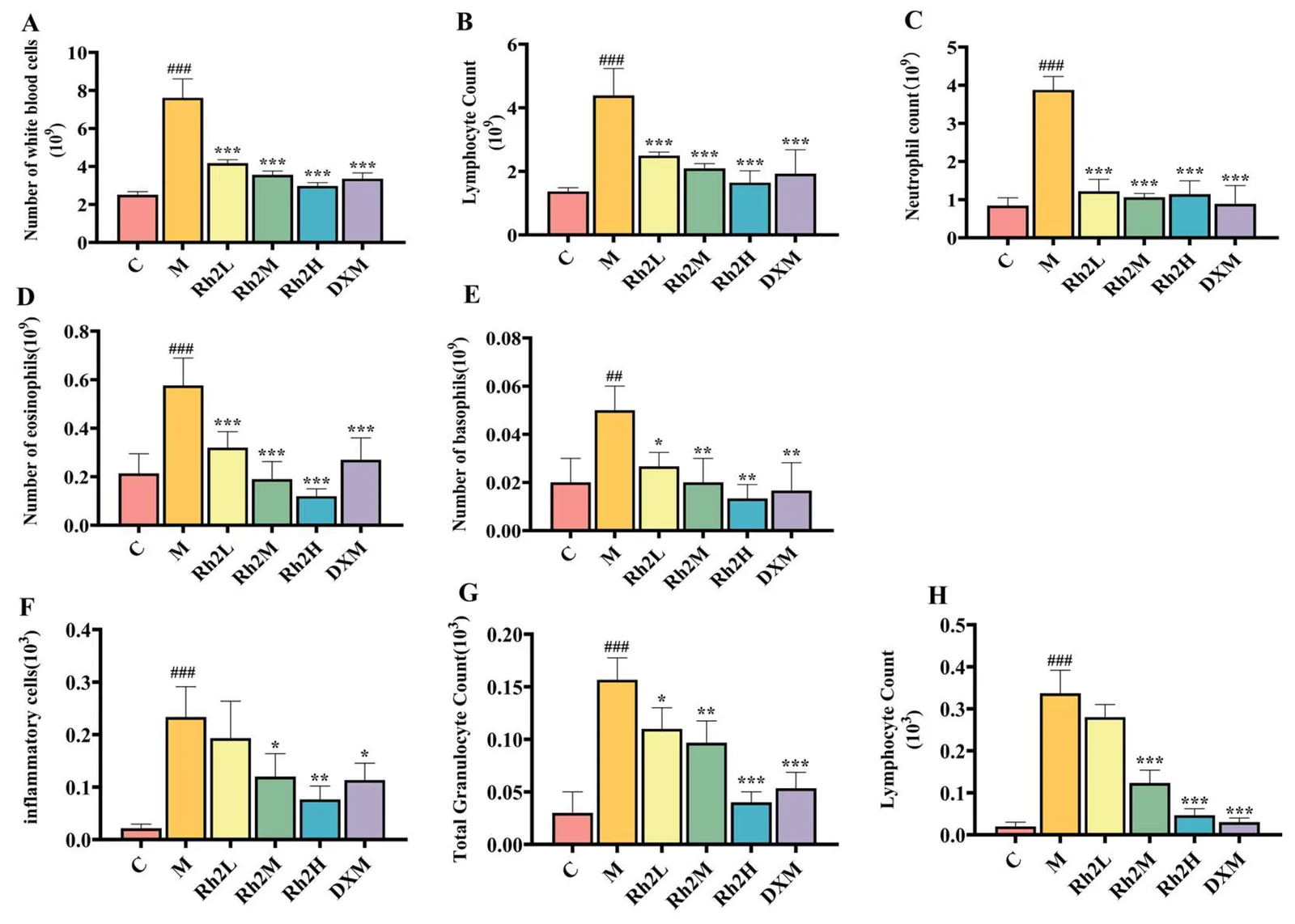

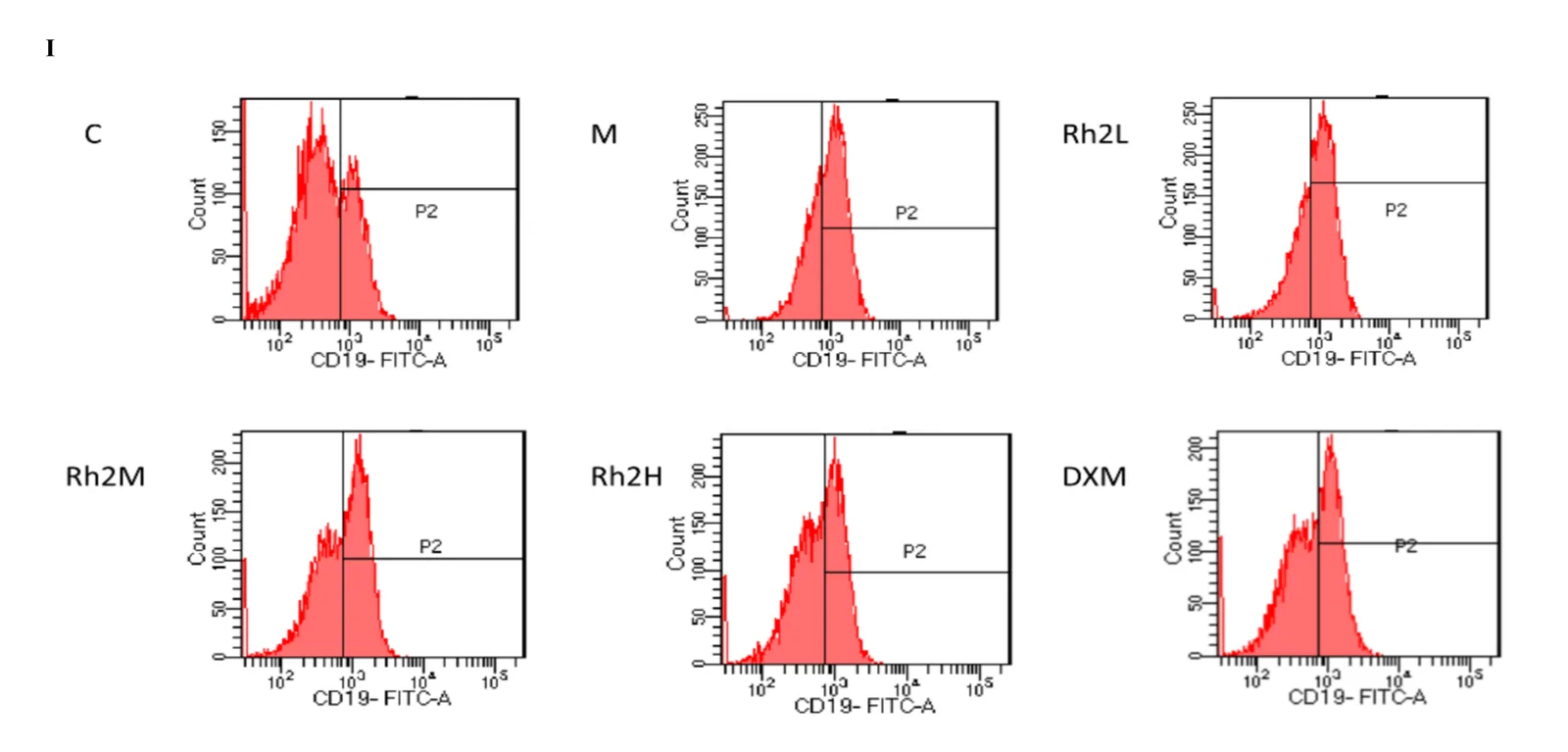
Effects of ginsenoside Rh2 on inflammatory-cell counts in peripheral blood and bronchoalveolar lavage fluid. White blood cells (**A**), lymphocytes (**B**), neutrophils (**C**), eosinophils (**D**), and basophils (**E**) were quantified in peripheral blood. Inflammatory cells (**F**), total granulocytes (**G**), and lymphocytes (**H**) were quantified in bronchoalveolar lavage fluid. The proportion of CD19-positive B cells in the spleen was analyzed by flow cytometry (**I**). Data are presented as mean ± SD (*n* = 3). ## *p* < 0.01, ### *p* < 0.001 vs. Group C; * *p* < 0.05, ** *p* < 0.01, *** *p* < 0.001 vs. Group M. One-way ANOVA followed by Tukey’s post hoc test was used for statistical analysis.

Peripheral blood cell analysis showed marked increases in white blood cells, lymphocytes, neutrophils, eosinophils, and basophils in the model group compared with the control group (Figure 5A–E; ### *p* < 0.001). Rh2 treatment significantly reduced these cell counts relative to the model group (*** *p* < 0.001).

Analysis of bronchoalveolar lavage fluid (BALF) revealed that the numbers of inflammatory cells, total granulocytes, and lymphocytes were significantly elevated in the model group (Figure 5F–H; ### *p* < 0.001), whereas Rh2 treatment significantly attenuated the elevation of all the above parameters (*** *p* < 0.001).

The proportion of CD19+ positive cells in the spleen was also examined by flow cytometry (Figure 5I). The model group showed an increased proportion of CD19+ positive cells compared with the control group, whereas Rh2 treatment reduced this proportion. These findings suggest that Rh2 alleviates systemic and pulmonary inflammatory-cell responses in the asthma model.

### 2.6. Effects of Ginsenoside Rh2 on Inflammatory Factors in Mice

**Figure 6:**
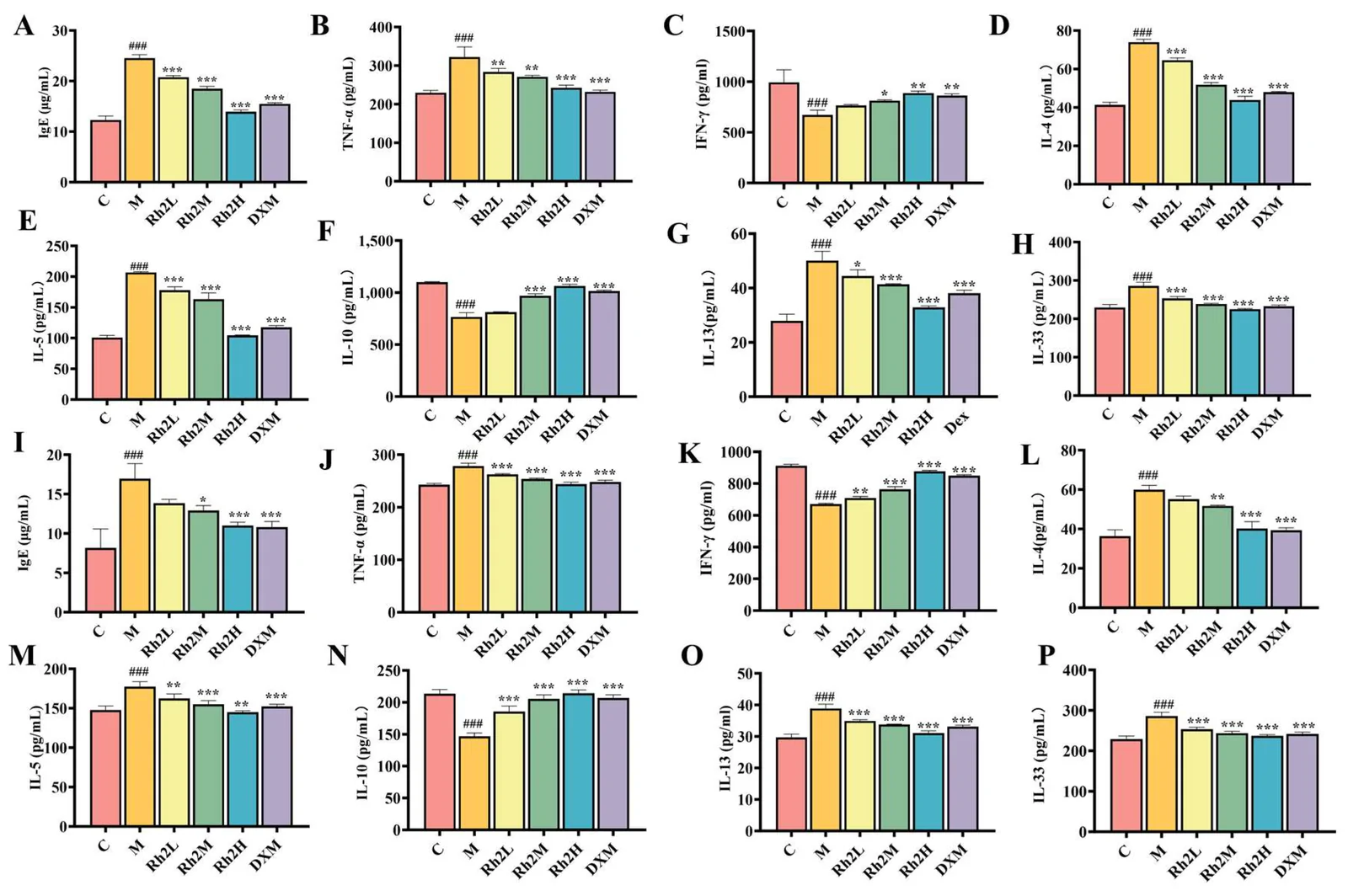
ELISA detection of cytokine levels (IgE, TNF-α, IL-4, IL-5, IL-13, IL-33, IFN-γ, and IL-10) in serum and alveolar lavage fluid of mice across groups. Rh2 treatment reduced serum levels of IgE (**A**), TNF-α (**B**), IL-4 (**D**), IL-5 (**E**), IL-13 (**G**), and IL-33 (**H**), while increasing levels of IFN-γ (**C**) and IL-10 (**F**). Rh2 treatment also decreased alveolar lavage fluid levels of IgE (**I**), TNF-α (**J**), IL-4 (**L**), IL-5 (**M**), IL-13 (**O**), and IL-33 (**P**), while elevating levels of IFN-γ (**N**) and IL-10 (**K**). Data are presented as mean ± SD (*n* = 3). ### *p* < 0.001 vs. Group C; * *p* < 0.05, ** *p* < 0.01, *** *p* < 0.001 vs. Group M. One-way ANOVA followed by Tukey’s post hoc test was used for statistical analysis.

Serum ELISA analysis revealed that, relative to the control group, the model group exhibited significantly elevated levels of IgE (Figure 6A), TNF-α (Figure 6B), IL-4 (Figure 6D), IL-5 (Figure 6E), IL-13 (Figure 6G), and IL-33/IL-13 (Figure 6H) (### *p* < 0.001), whereas the levels of anti-inflammatory factors IL-10 (Figure 6F) and IFN-γ (Figure 6C) were markedly reduced (### *p* < 0.001). In contrast, the ginsenoside Rh2 group showed significantly decreased secretion of these pro-inflammatory factors (*** *p* < 0.001) and significantly increased secretion of anti-inflammatory factors (*** *p* < 0.001) compared to the model group. These results indicate that ginsenoside Rh2 effectively regulates the balance between pro-inflammatory and anti-inflammatory factors in asthma mice, thereby mitigating the inflammatory response.

### 2.7. Regulatory Effects of Ginsenoside Rh2 on the Expression of Inflammation-Related Genes and Proteins in the Lung of Mice

**Figure 7:**
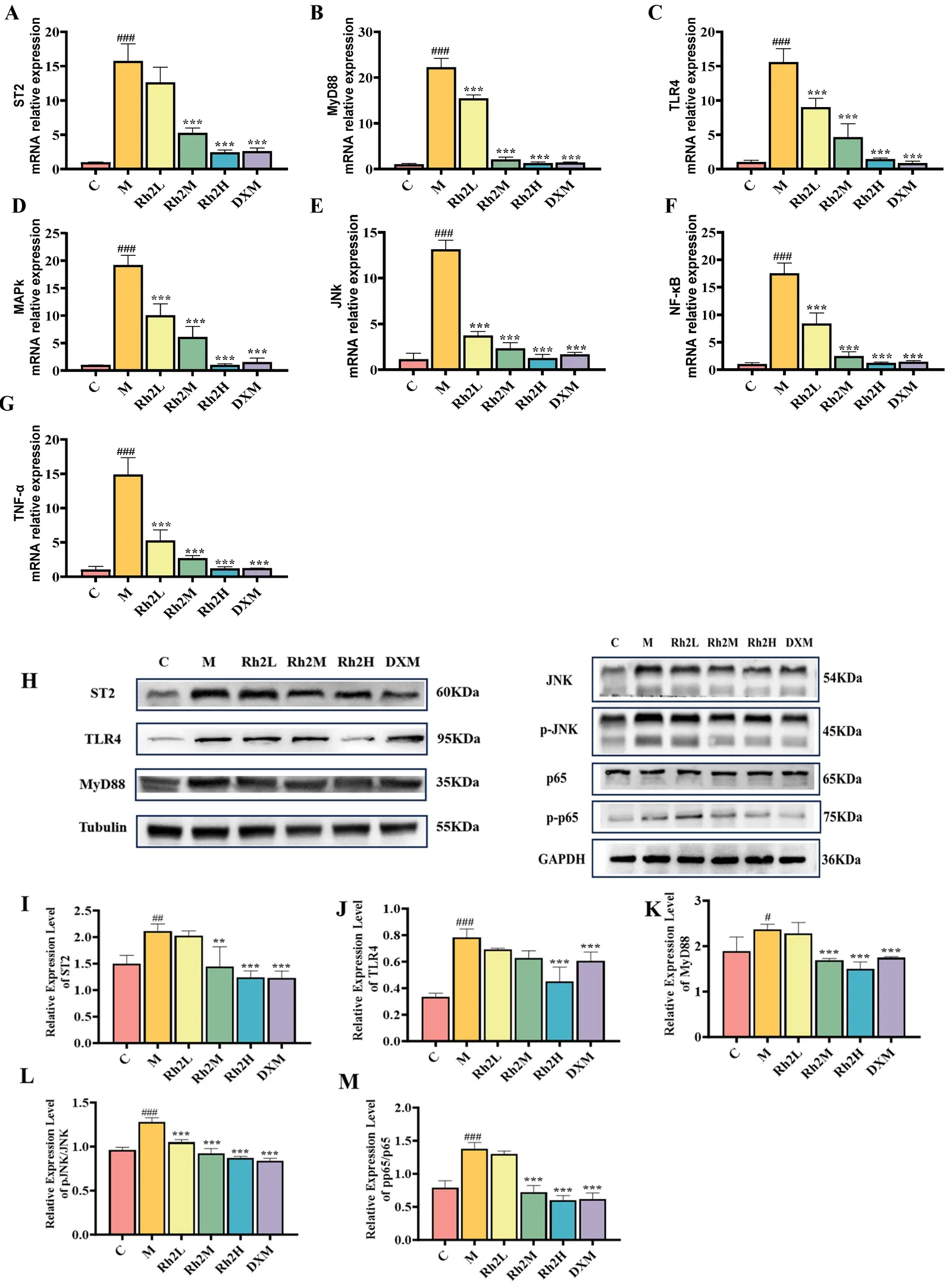

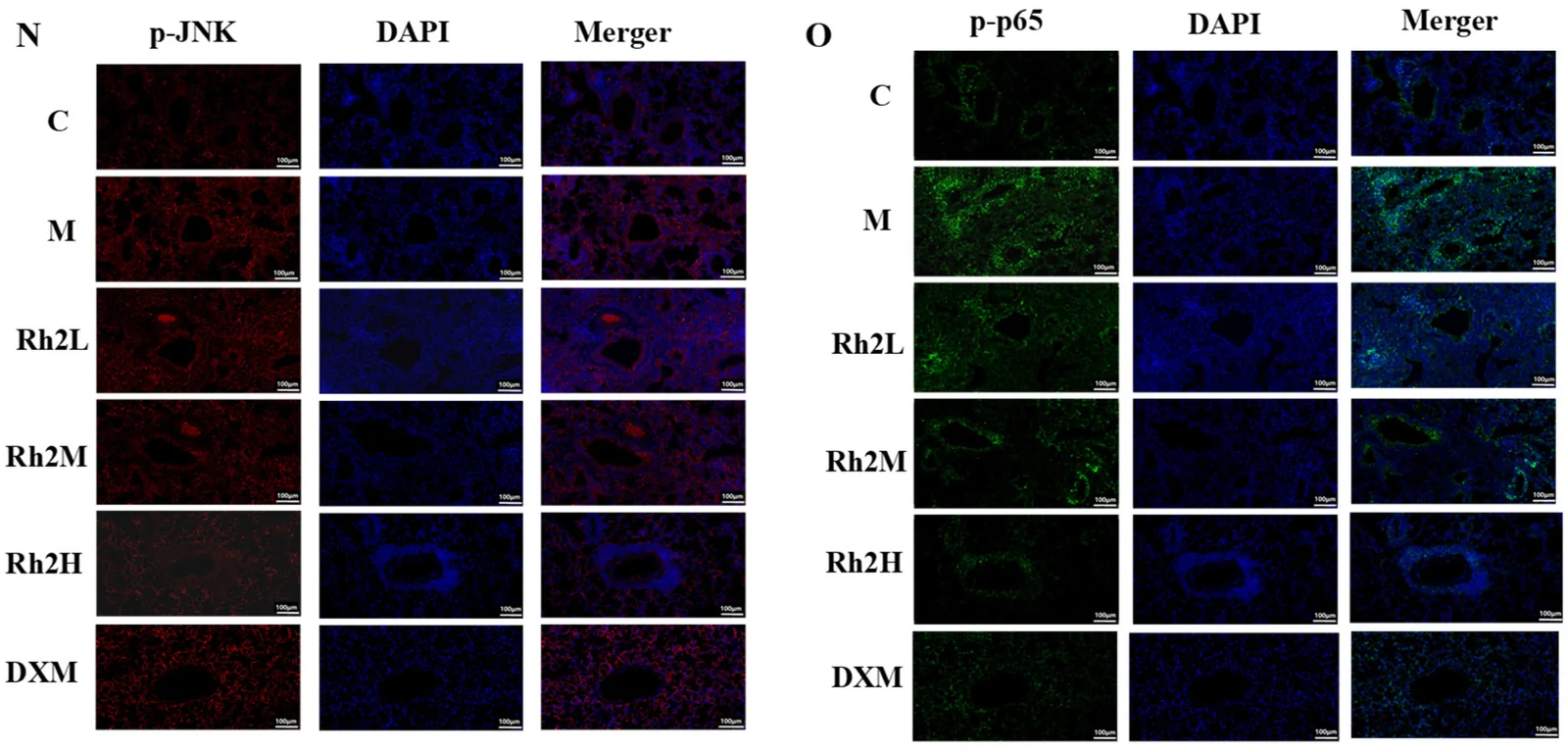
Effects of ginsenoside Rh2 on inflammatory signaling in mouse lung tissue. The mRNA expression levels of inflammation-related genes were determined by RT-qPCR (**A**–**G**). Protein expression levels of ST2, TLR4, MyD88, JNK, p-JNK, p65, and p-p65 were evaluated by Western blotting (**H**–**M**). Immunofluorescence staining was used to examine the expression and subcellular localization of p-JNK and p-p65 in lung tissue (**N**,**O**). Data are presented as mean ± SD (*n* = 3). # *p* < 0.05, ## *p* < 0.01, ### *p* < 0.001 vs. Group C; ** *p* < 0.01, *** *p* < 0.001 vs. Group M. One-way ANOVA followed by Tukey’s post hoc test was used for statistical analysis.

RT-qPCR analysis showed that the mRNA expression levels of ST2, MyD88, TLR4, and NF-κB were significantly increased in the model group compared with the control group (Figure 7A–D; ### *p* < 0.001). Treatment with Rh2 significantly reduced the expression of these genes (*** *p* < 0.001), suggesting that Rh2 may suppress inflammatory signaling associated with the TLR4 and NF-κB pathway.

The effects of Rh2 on inflammatory signaling were further assessed at the protein level. Western blot analysis showed that the model group had significantly increased levels of ST2, TLR4, MyD88, p-JNK, and p-p65 compared with the control group (Figure 7H–M; ### *p* < 0.001). Rh2 treatment significantly reduced the expression or phosphorylation levels of these signaling components relative to the model group (*** *p* < 0.001).

Immunofluorescence staining was subsequently performed to examine the intracellular distribution of p-JNK and p-p65 in lung tissues. In the model group, both p-JNK and p-p65 exhibited enhanced fluorescence signals with prominent nuclear localization. In contrast, Rh2 treatment reduced the fluorescence intensity of both proteins and was accompanied by a decrease in their nuclear localization (Figure 7N,O). These findings suggest that Rh2 exerts an inhibitory effect on the JNK and NF-κB-related inflammatory signaling pathways in the lungs of asthmatic mice. 

### 2.8. Regulatory Effect of Ginsenoside Rh2 on Gene-Transcription Levels in the Lung of Mice

**Figure 8:**
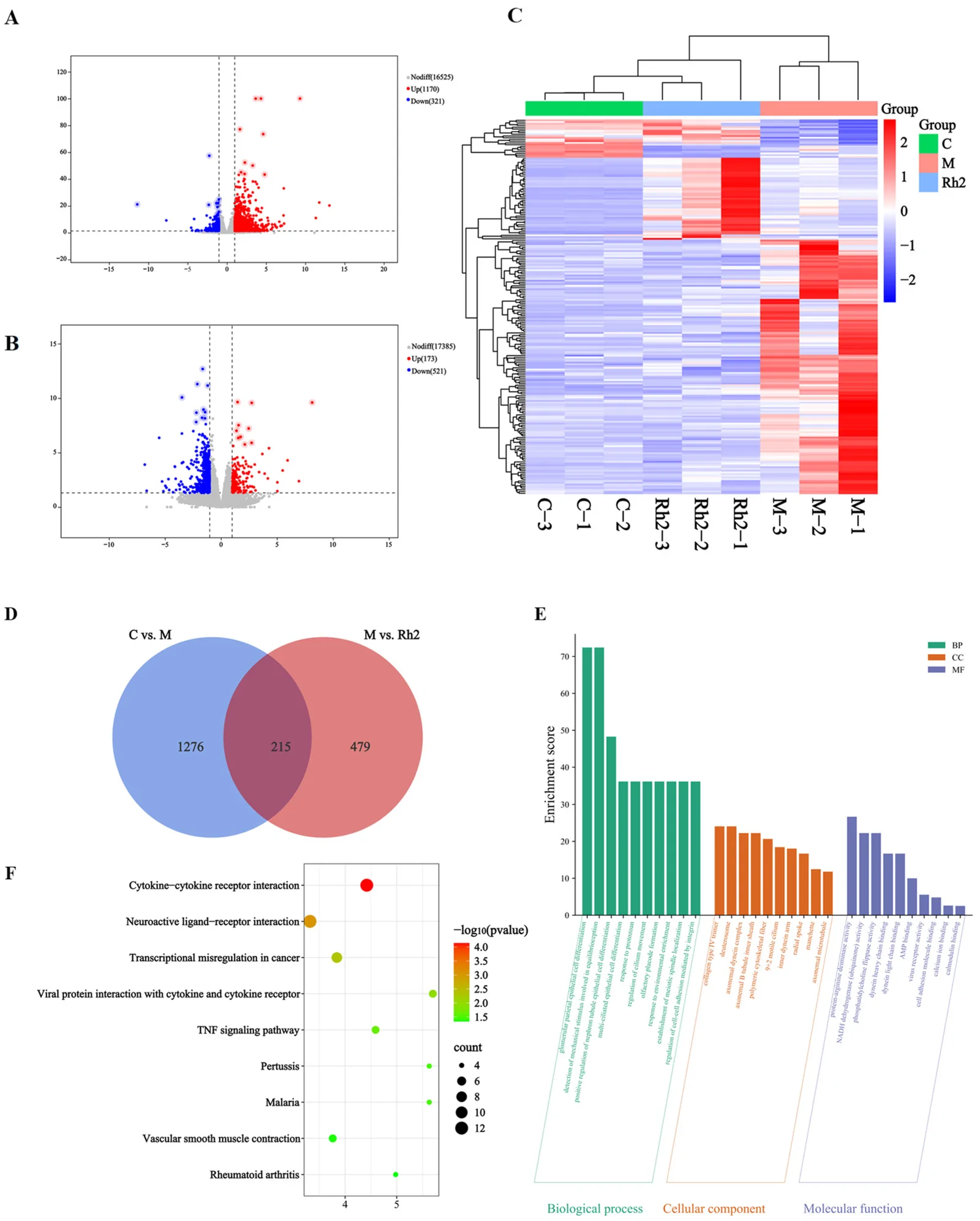
Volcano plot of differentially expressed genes (DEGs) in mice lungs: C vs. M group (**A**); volcano plot of DEGs in mice lungs: M vs. Rh2 group (**B**); Venn diagram of DEGs (**C**); hierarchical clustering heatmap of DEGs (**D**); GO functional analysis of DEGs (**E**); KEGG pathway enrichment analysis of DEGs (**F**).

RNA concentration analysis confirmed that the RNA samples met the requirements for sequencing (Supplementary Tables S3 and Supplementary Figure S3). This study first utilized a volcano plot to visually illustrate the distribution of differentially expressed genes. Comparison between Group C and Group M (Figure 8A), a total of 491 significantly differentially expressed genes were identified (upregulated: 170; downregulated: 321). Similarly, comparison between Group M and Group Rh2 (Figure 8B) revealed 694 significantly differentially expressed genes (upregulated: 173; downregulated: 521). Venn diagrams were constructed based on the sets of differentially expressed genes between Group C, Group M, Group Rh2 and Group M. The analysis identified 215 common genes shared between the comparisons of Group C with Group M and Group Rh2 with Group M (Figure 8D). To further investigate the effect of ginsenoside Rh2 on pulmonary gene-transcription levels in asthma model mice, cluster analysis and a heatmap were performed on these 215 common differentially expressed genes (results shown in Figure 8C). Clear separation trends were observed among Groups C, M, and Rh2, with Group Rh2 and Group C clustering together preferentially. These findings demonstrate that ginsenoside Rh2 effectively modulates dysregulated gene expression in the lungs of asthma model mice.

GO enrichment analysis was performed on 215 differentially expressed genes (results shown in Figure 8E). The results revealed that in terms of biological processes (BP), the genes were primarily enriched in immune defense-related processes such as positive regulation of eosinophil migration, leukocyte adhesion to vascular endothelial cells, and leukotriene signaling pathway; as well as in cellular structure and physiological homeostasis, and signal transduction, and neural regulation. In terms of cellular components (CC), the enrichment terms primarily include the Fc-epsilon receptor I complex, low-density lipoprotein particles, and the external surface of the plasma membrane—structures associated with cell junctions and physical boundaries, immune recognition and signaling complexes, as well as substance transport functions. Regarding molecular functions (MF), significant enrichment was observed in IgA binding, immunoglobulin receptor binding, and extracellular ligand-gated monoatomic ion channel activity, all of which are involved in immune system recognition and labeling, signal transduction, and channel-related cellular biological processes. These findings suggest that Rh2 may influence asthma progression by regulating cytoskeleton organization and protein modification processes.

To elucidate the biological functions of differentially expressed genes and the signaling pathways they participate in regulating, this study further conducted KEGG pathway enrichment analysis (results shown in Figure 8F). The results demonstrated that the differentially expressed genes were significantly enriched in multiple pathways related to asthma pathogenesis and immune regulation, including cytokine–cytokine receptor interaction, neuroactive ligand-receptor interaction, the tumor necrosis factor signaling pathway, and vascular smooth muscle contraction. These pathways may coordinate with each other to collectively form the therapeutic network of ginsenoside Rh2 in asthma treatment.

### 2.9. Regulation of Ginsenoside Rh2 on Metabolic Levels in the Lung of Mice

**Figure 9:**
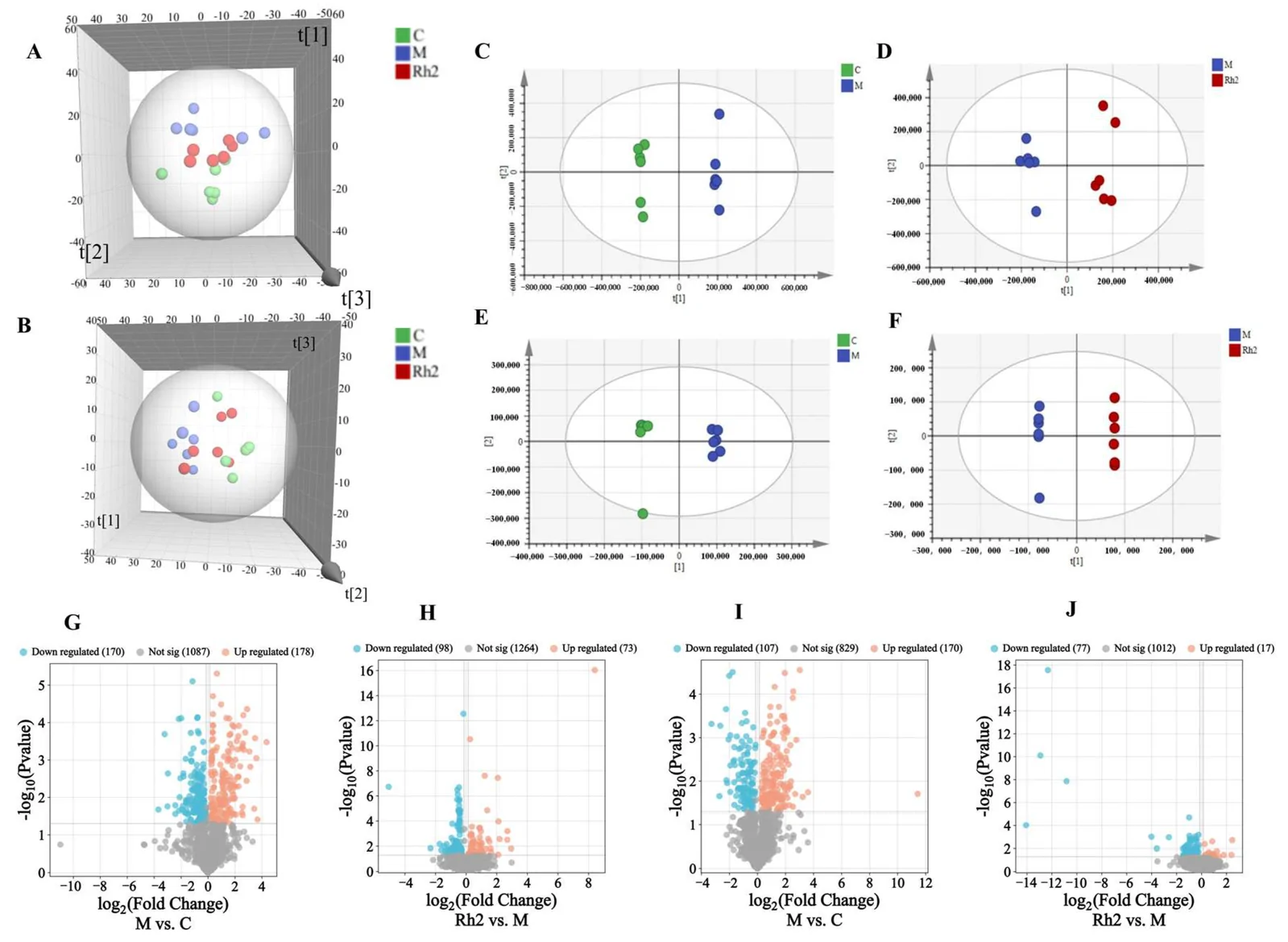

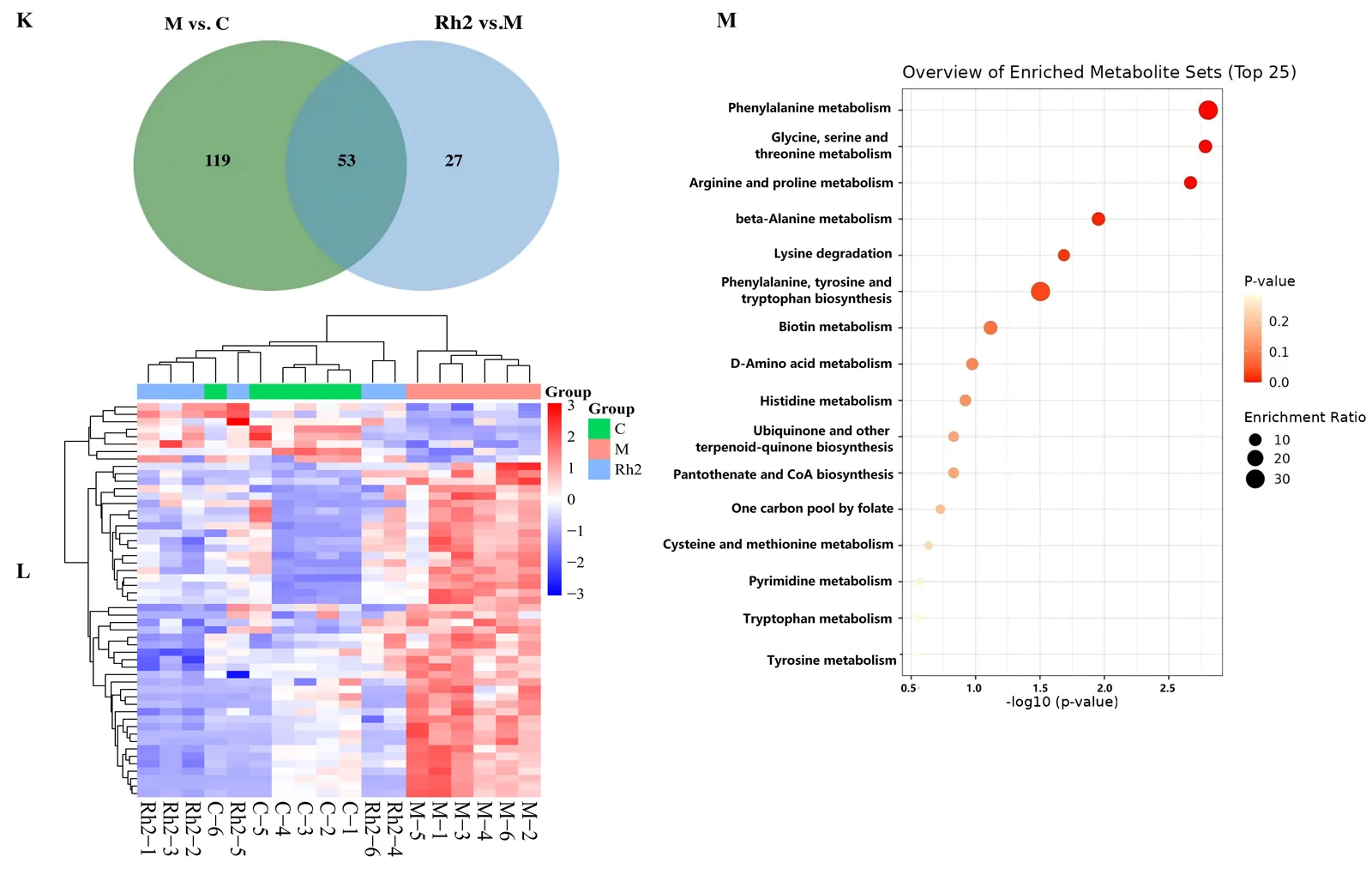
PCA and OPLS-DA plots of pulmonary metabolic profiling in mice across groups. PCA plot under positive ion mode (**A**); PCA plot under negative ion mode (**B**); OPLS-DA plot under positive ion mode (**C**,**D**); OPLS-DA plot under negative ion mode (**E**,**F**). Volcano plots of potentially differentially metabolized compounds in mice lungs across groups: positive spectrum C vs. M volcano plot (**G**) and M vs. Rh2 volcano plot (**H**); negative spectrum C vs. M volcano plot (**I**) and M vs. Rh2 volcano plot (**J**); Venn diagram of potentially differentially metabolized compounds in mice lungs (**K**); heatmap of differentially metabolized compounds in mice lungs (**L**); pathway map of differentially metabolized compounds in mice lungs (**M**).

PCA analysis of the pulmonary metabolomic data from each mice group (Figure 9A,B) revealed that samples from Group M and Group C exhibited significant spatial distance and markedly distinct intergroup differences. In contrast, samples from the treatment groups showed a clustering trend toward the control group. To further identify key differentially expressed metabolites, an OPLS-DA model was constructed (Figure 9C–F), demonstrating clear separation of samples from Group M from those of both Group C and the treatment groups. This finding further confirms the differences in metabolic profiles between Group M and the ginsenoside Rh2 group.

Before analyzing the significance of differential metabolites, we first examined data overfitting (for results see Supplementary Figure S4A,B). The volcano plot visually illustrates the statistical significance of metabolites and the magnitude of intergroup expression variations: in the positive spectrum comparing Group M with Group C, there were 178 upregulated and 170 downregulated differentially expressed metabolites (Figure 9G); in the negative spectrum, 170 were upregulated and 107 were downregulated (Figure 9H). Similarly, in the positive spectrum between Group Rh2 and Group M, 73 metabolites were upregulated and 98 were downregulated (Figure 9I), while in the negative spectrum, 17 were upregulated and 77 were downregulated (Figure 9J).

As a visualization tool capable of illustrating set overlap relationships, Venn diagrams clearly and intuitively demonstrate the intersection characteristics and intergroup differences among various datasets. In this study, screening thresholds were set at VIP > 1.0 (results shown in Supplementary Figure S3C,D), FC > 1.1 or <0.91, with *p* < 0.05. Differential metabolites were identified across different comparison groups in asthma mice and Venn diagrams were constructed accordingly (Figure 9K). A total of 172 differential metabolites were identified between Group M and Group C, while 80 differential metabolites were identified between Group Rh2 and Group M; 53 metabolites were shared between the two groups. Cluster analysis was performed on these shared differential metabolites, resulting in heatmap analysis (Figure 9L), which revealed clear separation trends among Groups C, M, and Rh2, with Group Rh2 and Group C predominantly clustered together.

To elucidate the signaling pathways regulated by differential metabolites, this study further conducted KEGG pathway enrichment analysis (Figure 9M). The results demonstrated that the differential metabolites were significantly enriched in amino acid metabolism-related pathways, including phenylalanine metabolism, glycine, serine, and threonine metabolism, as well as arginine and sulfoquinovose metabolism. These findings indicate that ginsenoside Rh2 can synergistically ameliorate metabolic abnormalities in asthma mice by modulating multiple metabolic pathways.

### 2.10. Association Analysis Between Differential Genes and Differential Metabolites

**Figure 10:**
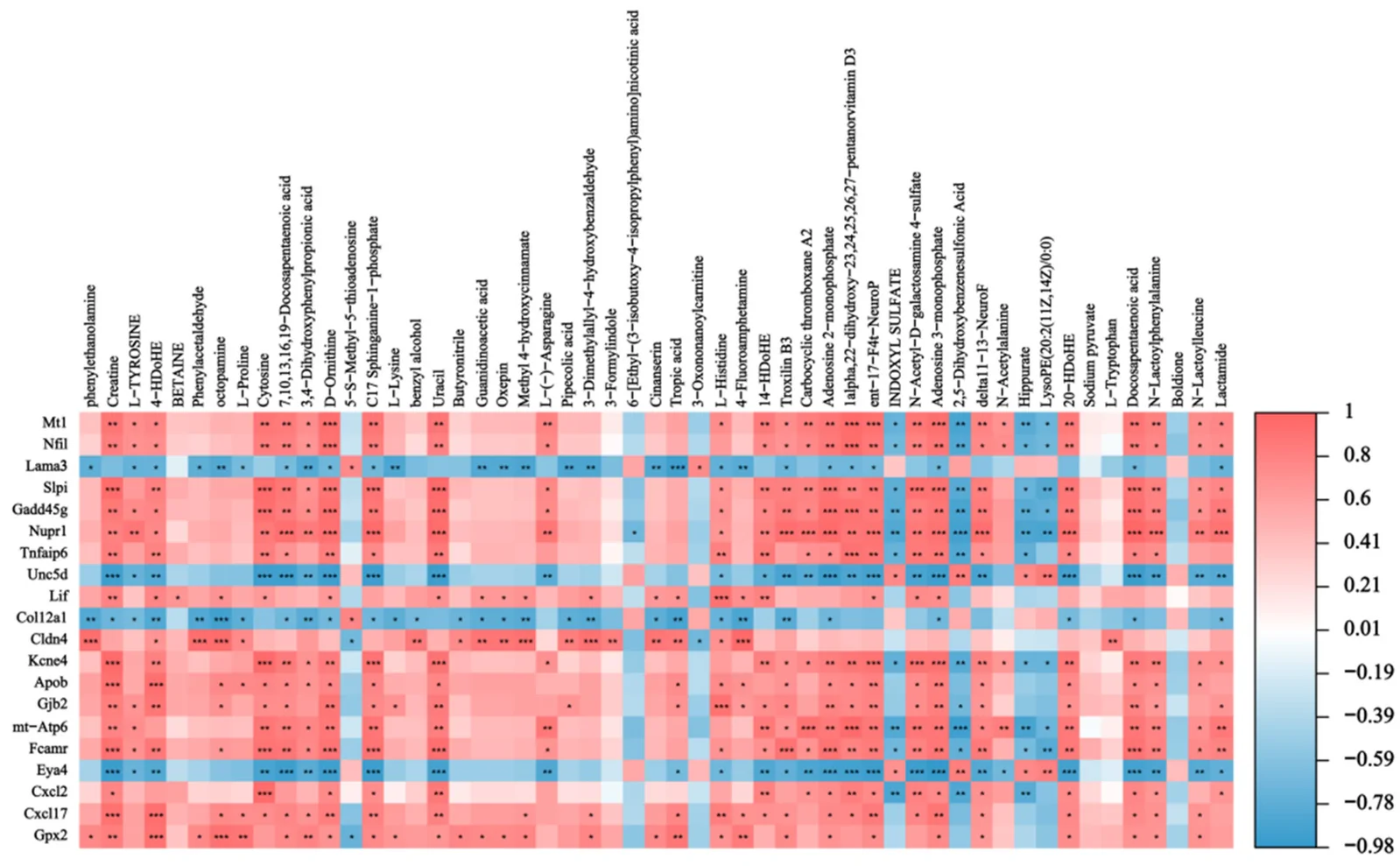
Heatmap showing associations between lung genes and metabolites in mice. (* *p* < 0.05, ** *p* < 0.01, *** *p* < 0.001, *n* = 3. One-way ANOVA, Tukey’s post hoc).

To investigate potential regulatory relationships between the transcriptome and metabolome, correlation analyses were performed between differentially expressed genes and differential metabolites, and correlation heatmaps were generated (Figure 10). *CXCL2* and *CXCL17* were positively correlated with cytosine (*** *p* < 0.01), whereas *LIF* was positively correlated with L-histidine (*** *p* < 0.01). These associations suggest potential links between transcriptional and metabolic alterations during asthma progression and Rh2 treatment.

## 3. Discussion

Cytokines, as key regulatory factors of the immune system, orchestrate intercellular interactions. Their functional dysregulation can drive the pathogenesis of chronic inflammation, cancer, and autoimmune diseases [16]. Based on their functional characteristics, cytokines are classified into three main categories: interleukins, chemokines, and growth factors. These molecules not only serve as biomarkers for disease progression but also represent clinically relevant potential therapeutic targets [17]. As core mediators of the innate immune system, the interleukin-1 (IL-1) family of cytokines not only dictates the course of acute and chronic inflammatory responses but also enhances the host’s non-specific resistance to infections [18]. This family occupies a unique hub position within the immune network: by promoting the maturation and activation of antigen-presenting cells, as well as regulating T-cell differentiation and B-cell responses, it effectively initiates specific immune defense mechanisms [19,20]. At the molecular level, IL-1 receptors share an intracellular Toll/interleukin-1 receptor (TIR) domain with Toll-like receptors (TLRs). This structural homology enables them to mimic TLR signaling cascades and activate downstream pathways. Consequently, this leads to the upregulation of adhesion molecules and the induction of pro-inflammatory cytokines and chemokines, thereby triggering robust inflammatory responses [21,22,23,24]. Therefore, the cytokine–cytokine receptor interaction pathway plays a pivotal role in the pathogenesis of asthma.

Bronchial epithelial cells lining the airway wall serve not only as a physical barrier but also play crucial roles in chemical defense, immune regulation, and injury repair. Under inflammatory conditions, elevated levels of inflammatory cytokines can induce epithelial–mesenchymal transition (EMT) in epithelial cells, promoting the deposition of collagen and fibronectin, which leads to basement membrane thickening [25]. Concurrently, inflammatory stimuli can drive the transdifferentiation of pseudostratified ciliated columnar epithelium into mucus-secreting goblet cells, thereby causing mucus hypersecretion [26]. Furthermore, Th1/Th2 imbalance exacerbates the release of inflammatory cytokines, leading to further damage of pulmonary airway cells. ELISA results demonstrated that ginsenoside Rh2 intervention significantly ameliorated the inflammatory response.

The IL-33/ST2, TLR4/MyD88/NF-κB, and MAPK/JNK signaling axes are classical inflammatory signaling pathways that play a central role in asthma pathogenesis. This study demonstrates that these signaling pathways are abnormally activated in asthma models, which may be closely associated with the sustained amplification of airway inflammation. IL-33, an “alarmitter” of the IL-1 family, is actively released by necrotic or damaged cells (e.g., epithelial and endothelial cells) and functions upstream in asthma pathogenesis. Upon binding to its receptor ST2L, IL-33 recruits the intracellular TIR domain to the adaptor protein MyD88, thereby converging and activating downstream NF-κB and MAPK pathways, driving the transcription and release of Th2-type inflammatory cytokines and exacerbating inflammatory responses in conditions such as allergies diseases, asthma, and atopic dermatitis. Notably, these three pathways share the adaptor protein MyD88 and their downstream effector molecules, forming a pivotal hub for inflammatory amplification. In resting conditions, NF-κB dimers bind to the inhibitory protein IκB and remain in the cytoplasm, thereby lacking transcriptional activity [27]. Under external stimuli such as OVA and LPS, the IκB kinase (IKK) complex is activated, leading to IκB phosphorylation and subsequent ubiquitination-mediated degradation. The released NF-κB translocates into the nucleus and undergoes phosphorylation modifications (e.g., p-p65), thereby promoting the production of pro-inflammatory cytokines.

Another major pathway in pulmonary transcriptome sequencing is the TNF-α signaling pathway. TNF-α exists in a homotrimeric form, comprising both transmembrane (tmTNF-α) and soluble (sTNF-α) isoforms. Both forms bind to the cell surface receptors Tumor Necrosis Factor Receptor 1 (TNFR1) and Tumor Necrosis Factor Receptor 2 (TNFR2), thereby triggering downstream signaling cascades that induce the release of pro-inflammatory cytokines and chemokines [28,29]. TNFR1 is widely expressed in nearly all human tissues and serves as the primary receptor mediating signal transduction [30,31]. Upon binding to TNFR1, TNF-α assembles into three distinct signaling complexes (complex I, II, and III), thereby regulating cellular fate [32]. The activation of complex I acts upstream to initiate key signaling pathways, such as NF-κB, MAPK, and AKT. In this study, key genes involved in the TNF-α pathway included *CXCL2*, *CXCL17*, and *LIF*. Previous research shows that elevated *CXCL2* gene expression recruits immunosuppressive neutrophils [33], while *CXCL17* regulates macrophage phagocytic function [16]; both *CXCL2* and *CXCL17* are closely associated with asthma exacerbation.

Analysis of pulmonary metabolites revealed that the primary metabolic pathways included pyrimidine metabolism, as well as glycine, serine, and threonine metabolism. Studies have demonstrated associations between amino acid levels and asthma status, and pulmonary function [34,35]. The metabolite cytosine is believed to enhance cellular immune responses. Human immune cells (e.g., B cells and dendritic cells) express a receptor called Toll-like receptor 9 (TLR9), which specifically recognizes unmethylated CpG sequences. Upon recognition of these “foreign” cytosine sequences, TLR9 triggers an alarm and activates the immune response, stimulating B-cell proliferation and antibody secretion to enhance the body’s resistance to bacteria and viruses. Serine and threonine are catalytically converted by specific enzymes into glycine, which serves as a precursor for creatine synthesis. The metabolite creatine alleviates cellular damage and inflammatory responses induced by excessive exercise [36]. Oxidative stress can form a positive feedback loop with NF-κB to amplify the inflammatory response [37]; the energy released during the breakdown of phosphocreatine—derived from creatine conversion—can convert adenosine diphosphate (ADP) and Pi back into adenosine triphosphate (ATP). Therefore, supplementation with creatine can alleviate oxidative stress, reduce ROS production, and ameliorate various diseases associated with mitochondrial dysfunction [38].

The association analysis between genes and metabolites revealed that the genes *CXCL2* and *CXCL17* exhibited positive correlations with the metabolite cytosine. This may be attributed to pyrimidine-mediated immune activation leading to CXCL2-mediated neutrophil recruitment to inflammatory sites to combat infection, while *CXCL17* promotes macrophage phagocytosis of harmful substances at inflammatory sites. Additionally, L-histidine showed a positive correlation with *LIF*. L-histidine is decarboxylated by histidine decarboxylase to form histamine; histamine is a bioactive substance that plays a crucial role in various physiological processes, including allergic reactions, inflammation, gastric acid secretion, and neural transmission. *LIF*, a pleiotropic cytokine belonging to the IL-6 family, is widely expressed in multiple tissues [39]. *LIF* can downregulate the expression levels of inflammatory factors; therefore, asthma mice may increase *LIF* secretion to suppress inflammation induced by L-histidine.

**Figure 11:**
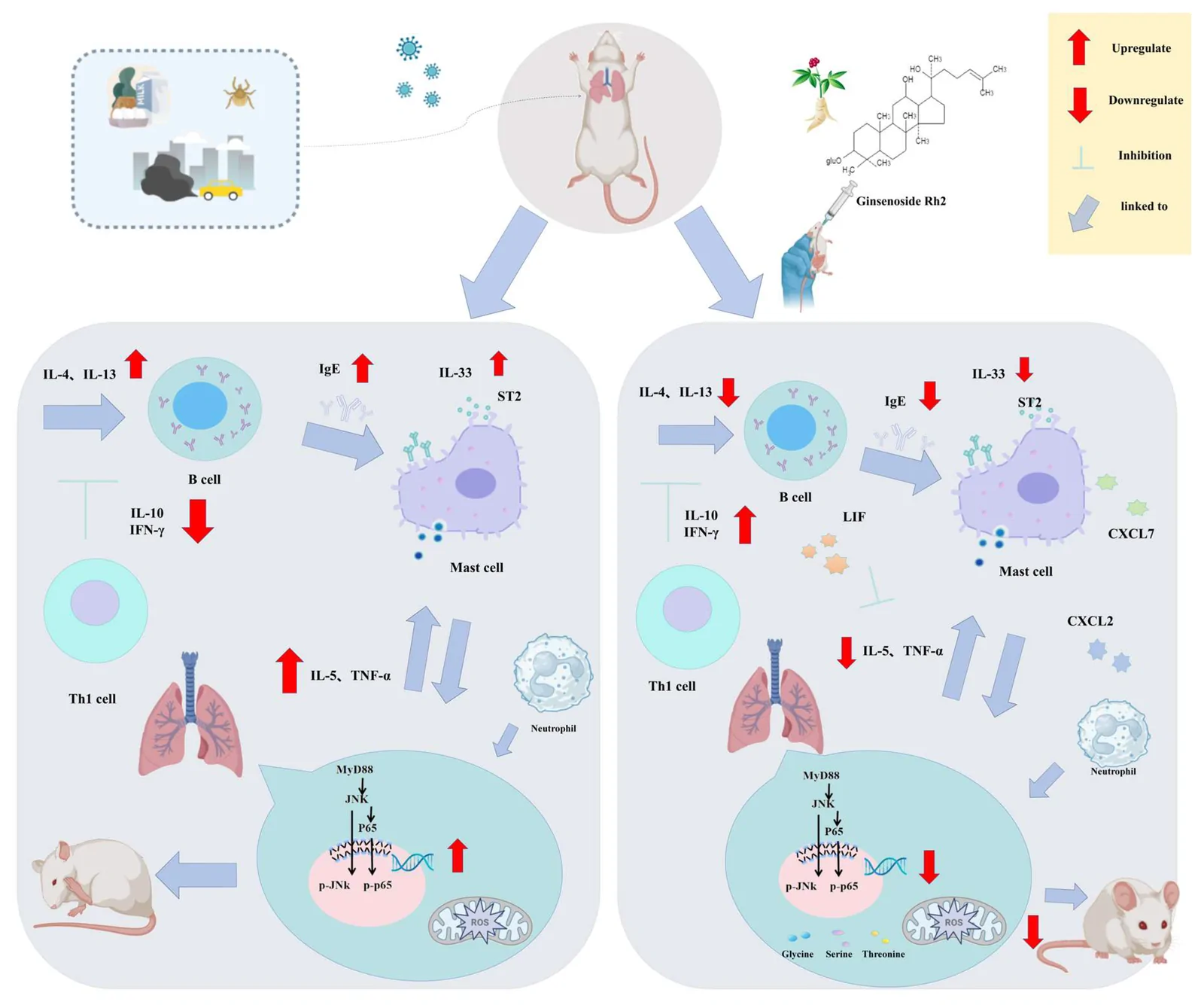
The mechanism of ginsenoside Rh2 in improving asthma.

The OVA-induced model primarily reflects allergic, type 2 immune-driven airway inflammation. Given that human asthma is a heterogeneous disease encompassing multiple phenotypes including eosinophilic, neutrophilic, and non-type 2 asthma, the findings of this study may not be directly applicable to all asthma phenotypes. Future studies utilizing alternative asthma models are warranted to determine whether the protective effects of Rh2 extend to other disease phenotypes. The results of this study indicate that Ginsenoside Rh2 can regulate the TNF-α signaling pathway, the cytokine–cytokine receptor interaction pathway, and various amino acid metabolic pathways. The synergistic effects among these pathways can effectively alleviate inflammatory responses, thereby improving asthma symptoms (Figure 11).

## 4. Materials and Methods

### 4.1. Reagents

LPS (cat: L8880) was purchased from Solabio Biotechnology Co., Ltd. (Beijing, China). The OVA (cat: A800) was purchased from Solabio Biotechnology Co., Ltd. (Beijing, China). Ginsenoside Rb1 (cat: B21061), ginsenoside Rg3 (cat: B21059), ginsenoside Rh2 (cat: B21062), ginsenoside Re (cat: B21055), ginsenoside Rg1 (cat: B21057), and ginsenoside Rh1 (cat: B21061) were purchased from Yuan Ye Biotechnology Co., Ltd. (Shanghai, China). Dexamethasone Acetate Tablets (cat: H41020216) were purchased from Changle Pharmaceutical Co., Ltd. (Xinxiang, China). Enzyme-linked Immunosorbent Assay (ELISA) Kit: Human IgE ELISA Kit [cat: ml105768], Human TNF-α ELISA Kit [cat: ml077385], Human IFN-γ ELISA Kit [cat: ml077386], Human IL-4 ELISA Kit [cat: ml058093], Human IL-5 ELISA Kit [cat: ml058095], Human IL-10 ELISA Kit [cat: ml064299], IL-13 ELISA Kit [cat: ml027429], Human IL-33 ELISA Kit [cat: ml027387], Mouse IgE ELISA Kit [cat: ml037602], Mouse TNF-α ELISA Kit [cat: ml002095], Mouse IFN-γ ELISA Kit [cat: ml002277], Mouse IL-4 ELISA Kit [cat: ml064310], Mouse IL-5 ELISA Kit [cat: ml002152], Mouse IL-10 ELISA Kit [cat: ml037888], Mouse IL-13 ELISA Kit [cat: ml037888], and Mouse IL-33 ELISA Kit [cat: ml063153] were purchased from Enzyme-linked Biotechnology Co., Ltd. (Shanghai, China). The total RNA extraction Kit (cat: ER501-01-V2) was purchased from Quanshijin Biotechnology Co., Ltd. (Beijing, China). The reverse transcription Kit (cat: RR047A) was purchased from TAKARA Biological Systems (Kyoto, Japan). The Mitochondrial Membrane Potential Assay Kit with JC-1 (cat: M8650) was purchased from Solabio Technology Co., Ltd. (Beijing, China). The Reactive Oxygen Species Assay Kit (cat: CA1410) was purchased from Solubio Technology Co., Ltd. (Beijing, China). The Anti-TLR4 Rabbit pAb (cat: GB11519-100), Anti-MyD88 Rabbit pAb (cat: GB111554-100), Anti-beta Tubulin Rabbit pAb (cat: GB11017), Anti-NF-κB p65 Rabbit pAb (cat: GB11997), Anti-Phospho-NF-κB p65 (cat: S536) Rabbit pAb (cat: GB113882), and Anti-GAPDH Rabbit pAb (cat: GB11002) were obtained from Severell Biotechnology Co., Ltd. (Wuhan, China). The IL-1RL1/ST2 Polyclonal Antibody (cat: GB11002) and Beta Actin Polyclonal Antibody (cat: 20536-1-AP) were sourced from Wuhan Sanying Biotechnology Co., Ltd. (Wuhan, China). The Anti-JNK1 + JNK2 + JNK3 Antibody (cat: ab179461) was purchased from Abcam plc (Cambridge, UK).

### 4.2. Experimental Animal

Specific pathogen-free (SPF) female BALB/c mice (9–11 weeks old; 20 ± 2 g) were purchased from Liaoning Changsheng Biotechnology Co., Ltd. (License No.: SCX (Liao) 2020-0001). All animal experiments were reviewed and approved by the Animal Ethics Committee of Changchun University of Chinese Medicine (Ethics Approval No.: 2025428), and all procedures were strictly conducted in accordance with the Regulations on the Management of Experimental Animals.

### 4.3. Establishment and Administration of the 16HBE Cell Inflammatory Model

The 16HBE cells were obtained from Shanghai Yaji Biological Technology Co., Ltd. The inflammatory-cell model was established by stimulation with LPS (10 μg/mL) for 16 h. The treatment groups included low-dose (Rh2L, 1 μg/mL), medium-dose (Rh2M, 5 μg/mL), and high-dose (Rh2H, 10 μg/mL) Rh2, as well as a dexamethasone-treated positive control group (DXM, 5 μg/mL). All cells were treated with the indicated compounds for 24 h.

### 4.4. Establishment of an Asthma Mice Model and Drug Administration

After 10 days of adaptive feeding, 36 mice were randomly divided into six experimental groups (*n* = 6 per group) as follows: control group (C), model group (M), low-dose Rh2 group (Rh2L, 2.5 mg/kg), medium-dose Rh2 group (Rh2M, 5 mg/kg), high-dose Rh2 group (Rh2H, 10 mg/kg), and dexamethasone positive control group (DXM, 1.25 mg/kg). Except for the C group, mice in all experimental groups received intraperitoneal injections of 0.2 mL of sensitizing solution (containing 10 μg OVA and 50 μL aluminum hydroxide adjuvant per mL) on days 10, 17, 24, and 31; the mice in C group received intraperitoneal injections of 0.2 mL of normal saline. From days 25 to 31, each mouse in the treatment groups received oral administration of the corresponding dose of ginsenoside Rh2. All groups underwent aerosol challenge with a 2.5% Ovalbumin (OVA) solution from days 29 to 31.

### 4.5. Detection of Mitochondrial Membrane Potential in 16HBE Cells

The 16HBE cells (2 × 10^5^ cells/well) were seeded in six-well plates and stimulated with LPS (10 μg/mL) for 16 h, followed by treatment with low-, medium-, or high-dose Rh2. The JC-1 working solution was prepared according to the Kit manufacturer’s instructions. After drug treatment, the culture medium was removed and the cells were washed once with Phosphate-Buffered Saline (PBS). Subsequently, 1 mL of fresh culture medium and 1 mL of JC-1 working solution were mixed, followed by incubation at 37 °C for 20 min in darkness. After incubation, the supernatant was discarded, and the cells washed twice with 1 × JC-1 staining buffer. Finally, 2 mL fresh culture medium was added, and fluorescence images were acquired using a fluorescence microscope (Nikon Instruments, Tokyo, Japan).

### 4.6. Detection of Reactive Oxygen Species Levels in 16HBE Cells

The 16HBE cells (2 × 10^5^ cells/well) were seeded in six-well plates and stimulated with LPS (10 μg/mL) for 16 h to establish the inflammatory model, followed by treatment with low-, medium-, or high-dose Rh2. After treatment, the medium was discarded, and 1 mL of DCFH-DA probe working solution (1:1000 dilution in serum-free medium) was added to each well. The cells were incubated at 37 °C for 20 min in the dark. After incubation, the probe solution was aspirated, and the cells were detached by pipetting with serum-free medium. The cell suspension was collected into centrifuge tubes and washed twice with serum-free medium, followed by centrifugation at 1000 rpm for 5 min. Subsequently, the supernatant was discarded, and the cells were resuspended in PBS for observation and imaging under a fluorescence microscope.

### 4.7. Behavioral Observation in Mice and Measurement of Airway Hyperresponsiveness

The weight of mice was first measured after three days of adaptive feeding, followed by weekly monitoring throughout the experimental period. For each measurement, weight was recorded three consecutive times, and the mean value was used for analysis. After the last OVA stimulation, the mice were placed individually in a transparent observation chamber for behavioral assessment. Clini symptoms and behavioral changes were observed and recorded, and a comprehensive scoring system was applied to each mouse based on the observed symptoms.

Acetylcholine chloride was dissolved in PBS to prepare a 6.5 mg/mL solution. Each mouse was placed in the body scanning chamber and allowed to acclimate for 15 min, followed by recording the baseline value of the exhalation interval (Penh) over approximately 3 min. Subsequently, 150 μL of PBS was added to the nebulizer and nebulized for 1 min, and the Penh value was recorded within 3 min after inhalation of the solvent. After a 4 min stabilization period, 150 μL of the acetylcholine chloride solution (6.5 mg/mL) was added to the nebulizer for a 1 min inhalation challenge, with the values recorded during the subsequent 3 min.

### 4.8. Histological Staining of Mouse Lung Tissue

Mice were randomly assigned to different experimental groups. Blinding was not performed during the intervention phase of this study. After the mice were sacrificed, lung tissues from each group were randomly sampled for subsequent assays. After dewaxing and hydration of the embedded lung tissue sections, conventional staining was performed using H&E, PAS, and Masson’s trichrome staining methods. Subsequently, the sections were then mounted with neutral gum for preservation. Histopathological changes in lung tissue samples from each group were observed under a microscope.

### 4.9. Enumeration of Inflammatory Cells in Blood and BALF

Mice were anesthetized with chloral hydrate, and blood samples were collected via the retro-orbital bleeding method. A midline cervical incision was made to expose the trachea, followed by thoracotomy to expose the lungs. The left main bronchus was then ligated. Subsequently, 0.5 mL of pre-cooled PBS was injected via a catheter for alveolar lavage, and the lavage fluid was collected into a centrifuge tube. The collected bronchoalveolar lavage fluid (BALF) was centrifuged at 4 °C and 1000 rpm for 10 min. The cell pellet was resuspended in 1 mL of PBS, and cellular analysis was performed using a hematology analyzer (Sysmex Corporation, Kobe, Japan) on both blood and BALF samples.

### 4.10. ELISA for Detecting Inflammatory Cytokine Levels in 16HBE Cell Supernatant, Serum, and BALF

The 16HBE cells were seeded in 6-well plates and treated with the indicated compounds. After treatment, the culture supernatants were collected and centrifuged at 3000 rpm for 10 min to remove cellular debris. According to the ELISA Kit instructions, the concentrations of IgE, TNF-α, IFN-γ, IL-4, IL-5, IL-10, IL-13, and IL-33 in the supernatants were measured.

Mice were anesthetized with chloral hydrate, and blood samples were collected via retro-orbital bleeding. Blood samples collected via the retro-orbital route were allowed to stand for 30 min and then centrifuged at 3500 rpm for 20 min. BALF was centrifuged at 1000 rpm for 10 min at 4 °C. The supernatants from both samples were collected and stored at −20 °C for further analysis. The detection of factors in both serum and BALF was performed as described above.

### 4.11. Flow Cytometry Detection of B Cells in Mice Spleen

The mice spleen was mechanically dissociated and rinsed with PBS to obtain the cell suspension into a centrifuge tube. The suspension was centrifuged at 2000 rpm for 5 min at 4 °C. The supernatant was discarded, and the cell pellet was resuspended in 4 mL of erythrocyte lysis solution. The mixture was incubated on ice for 5 min to lyse the erythrocytes, followed by another centrifugation at 4 °C and 2000 rpm for 5 min. The supernatant was discarded, and the cells were resuspended in fresh medium or buffer and adjusted to a density of 1 × 10^7^ cells/mL. One mL of the cell suspension was transferred to a 24-well plate and stimulated with 10 μL each of phorbol ester (PMA, final concentration 25 ng/mL) and ionomycin (final concentration 1 mg/mL) at 37 °C for 30 min. Subsequently, 20 μL of brefeldin A (5 mg/mL) was added, and the stimulation continued for 3.5 h. After stimulation, the cells were stained using PE anti-mice CD19 antibody (E-AB-F0986D, Elabscience, Wuhan, China) according to the manufacturer’s instructions, followed by flow cytometric analysis.

### 4.12. Detection of Expression Levels of Inflammation-Related Genes

The expression levels of inflammation-related genes in 16HBE cells were quantified by RT-qPCR. Total RNA was extracted according to the manufacturer’s instructions using a total RNA extraction Kit (Product No.: ER501-01-V2, TransGen Biotech, Beijing, China).

The expression levels of inflammation-related genes in mice lung tissues were detected using RT-qPCR. Frozen lung tissues were rapidly ground into powder in a mortar pre-cooled with liquid nitrogen. The tissue powder was transferred to centrifuge tubes, followed by the addition of 1 mL TransZol Up reagent and 0.2 mL chloroform. The mixture was vortexed vigorously until no visible precipitate remained, and total RNA was extracted according to the manufacturer’s protocol.

Reverse transcription was performed using the PrimeScript™ RT Reagent Kit with gDNA Eraser (Takara Bio, Kyoto, Japan) according to the manufacturer’s instructions. The relative mRNA expression levels were calculated using the 2^−ΔΔCt^ method.The gene sequences of the cells are listed in Table 1, and those of the mice are listed in Table 2.

**Table 1:** Primers used for real-time quantitative PCR analysis in cells.

*Gene Name*	Forward (5′ → 3′)	Reverse (5′ → 3′)
*ST2*	GGCGGCTTGCAGAGATACTT	GCGGTAGAGGATGTTGAGCAC
*MyD88*	GGCTGCTCTCAACATGCGA	CTGTGTCCGCACGTTCAAGA
*TLR4*	TTTGGACAGTTTCCCACATTGA	AAGCATTCCCACCTTTGTTGG
*MAPK*	CGGTGTCAATGGTTTGGTGC	GACGATGTTGTCGTGGTCCA
*JNK*	GGGTATGCCCAAGAGGACAGA	GTGTTGGAAAAGTGCGCTGG
*NF-κB*	TCCATATTTGGGAAGGCCTGAA	TTGAAGGTATGGGCCATCTGT
*TNF-α*	ATCGGTCCCCAAAGGGATGA	GGAGGTTGACCTTGGTCTGG
*GAPDH*	GGAGCGAGATCCCTCCAAAAT	GGCTGTTGTCATACTTCTCATGG

**Table 2:** Primers for real-time quantitative PCR analysis of mouse lung.

Gene Name	Forward (5′ → 3′)	Reverse (5′ → 3′)
*ST2*	CAAGGTTCAGGGCACACAGGTC	GCCGTCACGATGTAGTTGGTTCC
*MyD88*	ATCGCTGTTCTTGAACCCTCG	CTCACGGTCTAACAAGGCCAG
*TLR4*	AACCTGCTCTACCTACACCTG	CCGAGAGATTGAGGAATCGAAG
*MAPK*	GCAACCTCGCTGTGAATGAAGA	CACGTAGCCGGTCATTTCGTC
*JNK*	AGCCTGCCTGCCAAGTACAAG	TCAGGTTGACACCAGCAGCAC
*NF-κB*	GGGGCCTGCAAAGGTTATC	TGCTGTTACGGTGCATACCC
*TNF-α*	CCCTCACACTCACAAACCAC	ACAAGGTACAACCCATCGGC
*GAPDH*	AGGTCGGTGTGAACGGATTTG	GGGGTCGTTGATGGCAACA

### 4.13. Western Blot Analysis of Inflammatory Signaling Proteins in Mouse Lung Tissue

Approximately 80 mg of lung tissue was homogenized in 1 mL of pre-cooled total protein extraction buffer. The homogenate was kept on ice for 30 min and then centrifuged at 14,000 rpm for 10 min at 4 °C. The resulting supernatant was collected for protein analysis. Equal amounts of protein were separated by SDS-PAGE using a 10% resolving gel and subsequently transferred onto polyvinylidene difluoride (PVDF) membranes. The membranes were blocked with rapid blocking solution for 15 min at room temperature and then incubated with primary antibodies diluted in antibody dilution buffer overnight at 4 °C. Antibody information is listed in Table 3. After washing three times with TBST, the membranes were incubated with the corresponding secondary antibodies for 1 h at room temperature. The membranes were washed again with TBST, and protein signals were visualized using a chemiluminescence imaging system (Bio-Rad Laboratories, Hercules, California, USA). Band intensities were quantified using ImageJ software (ImageJ version 1.54, National Institutes of Health, Bethesda, MA, USA).

### 4.14. Immunofluorescence Analysis in 16HBE Cells and Mouse Lung Tissue

For cell immunofluorescence staining, slide-mounted cells were permeabilized with 0.5% Triton X-100 for 30 min at room temperature and washed three times with PBS for 3 min each. The cells were then blocked with 5% goat serum for 30 min at room temperature and incubated with the appropriate primary antibodies overnight at 4 °C. The following day, the cells were washed with PBS and incubated with fluorescently labeled secondary antibodies for 1 h at room temperature in the dark. After three additional washes with PBS, the nuclei were stained with DAPI for 5 min under light-protected conditions. The slides were mounted with an anti-fade mounting medium and examined using a fluorescence microscope.

For lung tissue immunofluorescence staining, paraffin sections were deparaffinized with xylene and rehydrated through a graded ethanol series. Antigen retrieval was performed using citrate buffer and microwave heating. After cooling to room temperature, the sections were washed with PBS and blocked with 5% BSA for 40 min. Primary antibodies diluted in PBS containing 1% BSA were applied to the sections, followed by overnight incubation at 4 °C in a humidified chamber. The sections were washed with PBS, counterstained with DAPI, mounted, and imaged using a fluorescence microscope.

### 4.15. RNA Extraction from Mice Lungs, Library Construction, Sequencing, and Sequence Alignment

Total RNA was isolated from mouse lung tissue using a magnetic bead-based extraction method. RNA quality was assessed before library preparation. Sequencing libraries were constructed using the NEBNext Ultra II RNA Library Prep Kit for Illumina (New England Biolabs, Ipswich, MA, USA) and evaluated using an Agilent 2100 Bioanalyzer (Agilent Technologies, Santa Clara, CA, USA). Qualified libraries were normalized, pooled, and sequenced on an Illumina platform using paired-end 150 bp reads. The reference genome and corresponding gene annotation files were obtained from the NCBI database. HISAT2 (v2.1.0) was used to construct the reference genome index and align the paired-end clean reads to the reference genome.

### 4.16. Gene Expression Levels in Mice Lungs and Bioinformatics Analysis of Differentially Expressed Genes

Gene-level read counts were generated using HTSeq (v0.9.1). Gene expression was normalized as fragments per kilobase of transcript per million mapped reads (FPKM) to account for differences in gene length and sequencing depth.

Differentially expressed genes (DEGs) were identified using the criteria of |log2FC| > 1 and *p* < 0.05. Volcano plots and hierarchical clustering heatmaps were generated to visualize expression differences among groups. Functional annotation and pathway enrichment analyses were performed using DAVID for Gene Ontology (GO) and Kyoto Encyclopedia of Genes and Genomes (KEGG) analyses. Graphical visualization was generated using the MicroBioinformatics Cloud Platform.

### 4.17. Extraction and Detection of Metabolites from Mice Lung Tissues

Mouse lung tissue samples were homogenized using a high-throughput tissue homogenizer(Shanghai Jingxin Industrial Development Co., Ltd., Shanghai, China). An 800 μL aliquot of pre-cooled methanol/acetonitrile solution (1:1, *v*/*v*) was added to each sample, followed by ultrasonic extraction for 30 min in an ice-water bath. The extracts were centrifuged at 12,000 rpm for 10 min at 4 °C. The supernatants were collected and dried under vacuum. The residues were reconstituted in 150 μL of 50% methanol containing 5 ppm 2-chlorophenylalanine and vortexed for 30 s. After centrifugation at 12,000 rpm for 10 min at 4 °C, the supernatants were filtered through 0.22 μm organic-phase filters prior to LC-MS analysis.

Metabolite separation was performed using an ACQUITY UPLC HSS T3 column (Waters, Milford, MA, USA, 100 Å, 1.8 μm, 2.1 mm × 100 mm). The flow rate was 0.4 mL/min, the column temperature was maintained at 40 °C, and the autosampler temperature was set to 8 °C. The injection volume was 2 μL. Mobile phase A consisted of water containing 0.1% formic acid, whereas mobile phase B consisted of acetonitrile containing 0.1% formic acid. The gradient program is presented in Table 4.

Data-dependent acquisition was performed using a Thermo Orbitrap Exploris 120 mass spectrometer (Thermo Fisher Scientific, Waltham, MA, USA) equipped with an HESI source and controlled by Xcalibur 4.7 software. The spray voltage was set to 3.5 kV in positive ion mode and −3.0 kV in negative ion mode. The sheath and auxiliary gas flow rates were set to 40 and 15 arbitrary units, respectively. The capillary temperature was maintained at 325 °C, and the auxiliary gas heater temperature was set to 300 °C. Full-scan MS data were acquired at a resolution of 60,000 over an *m*/*z* range of 100–1000. The four most intense precursor ions were selected for fragmentation. MS/MS spectra were acquired at a resolution of 15,000 using higher-energy collisional dissociation with a normalized collision energy of 30%.

### 4.18. Metabolite Profiling and Identification of Differential Metabolites

Raw LC-MS data were processed using Compound Discoverer 3.3 software (version 3.3.2.31; Thermo Fisher Scientific, Waltham, MA, USA). Signal intensities were normalized using the sum normalization method. Metabolites were annotated by searching the acquired spectra against an in-house database, mzCloud, LIPID MAPS, HMDB, MoNA, and the NIST 2020 MS/MS spectral library. Metabolite identification was performed using a precursor mass tolerance of 15 ppm and a fragment match factor of at least 50.

Potential differential metabolites were screened using the criteria of VIP > 1, *p* < 0.05, and FC > 1.1 or FC < 0.91. Heatmaps and metabolic pathway enrichment analyses were generated using MetaboAnalyst 6.0.

### 4.19. Data Processing and Statistical Analysis

The experimental data were plotted using GraphPad Prism 8.0 software and statistically analyzed by one-way analysis of variance (ANOVA) and Dunnett’s multiple comparison tests. Statistical differences were expressed as follows: compared with Group C, # *p* < 0.05, ## *p* < 0.01, ### *p* < 0.001; compared with Group M, * *p* < 0.05, ** *p* < 0.01, *** *p* < 0.001.

## Figures and Tables

**Table 3:** Antibody information.

Antibody	Catalog No.	Dilution
ST2	GB11002	1:1000
MyD88	GB111554-100	1:1000
TLR4	GB11519-100	1:1000
JNK	ab179461	1:1000
p-JNK	80024-1-RR	1:2000
p65	GB11104	1:1000
p-p65	GB113882	1:500
Tubulin	GB11017	1:1000
GAPDH	GB11002	1:1000

**Table 4:** Chromatographic methods.

Time (min)	B%
0	5%
1	5%
7	95%
8	95%
8.1	5%
12	5%

## Data Availability

The original contributions presented in this study are included in the article/Supplementary Materials. Further inquiries can be directed to the corresponding author(s).

## Supplementary Materials

The following supporting information can be downloaded at https://www.mdpi.com/article/10.3390/ijms27188160/s1.
