# Supplementary material for: Study on the Therapeutic Effects of Ginsenoside Rh2 in Asthma Improvement
Source: Int J Mol Sci. 2026 Sep 14;27(18):8160. doi: 10.3390/ijms27188160 (PMC13607476; doi:10.3390/ijms27188160)
Supplement: Supplementary file 1 [file ijms-27-08160-s001.zip › ijms-4493543-supplementary.pdf]

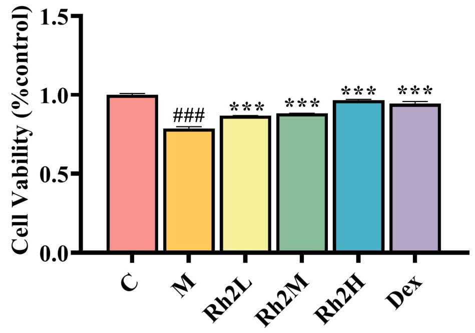

Supplementary Fig S1. Cell viability assessment of each group after modeling and drug administration.

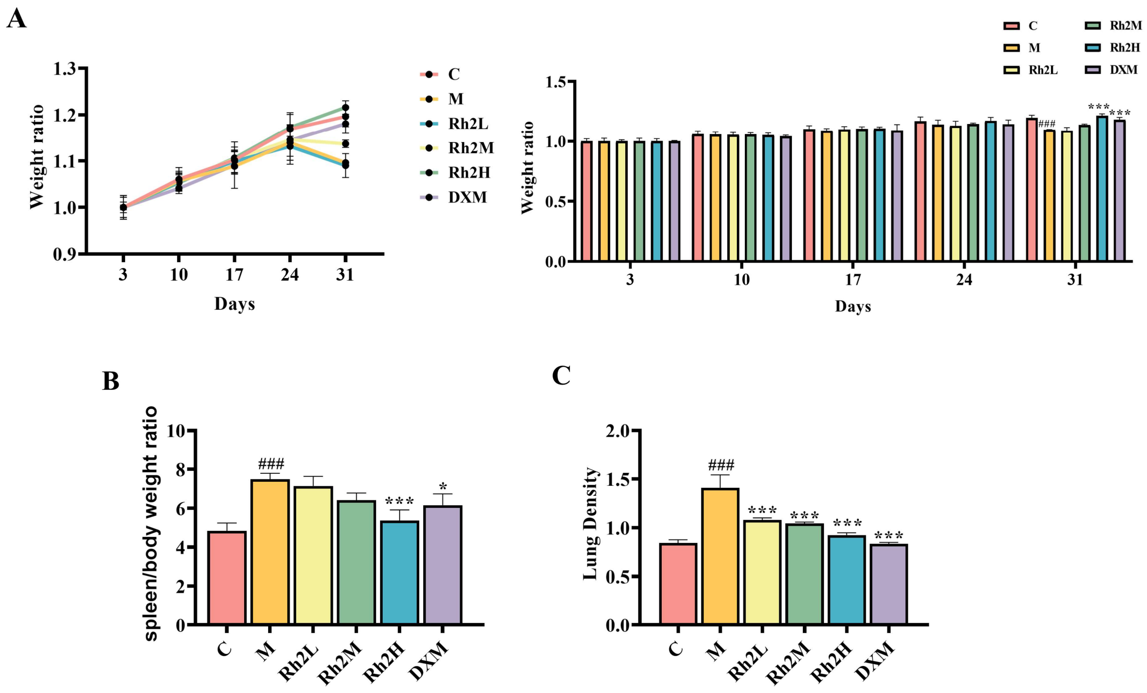

Supplementary Fig S2: Effects of ginsenoside Rh2 on body weight（A）, spleen-to-body weight ratio（B）, and lung-to-body weight ratio in mice（C）. (Compared with Group C, $^{\#\#\#}p$<0.001; compared with Group M, $^{*}p$<0.05, $^{**}p$<0.01, $^{***}p$<0.001, values are mean ± SD, *n*=3. One-way ANOVA, Tukey’ s post hoc.).

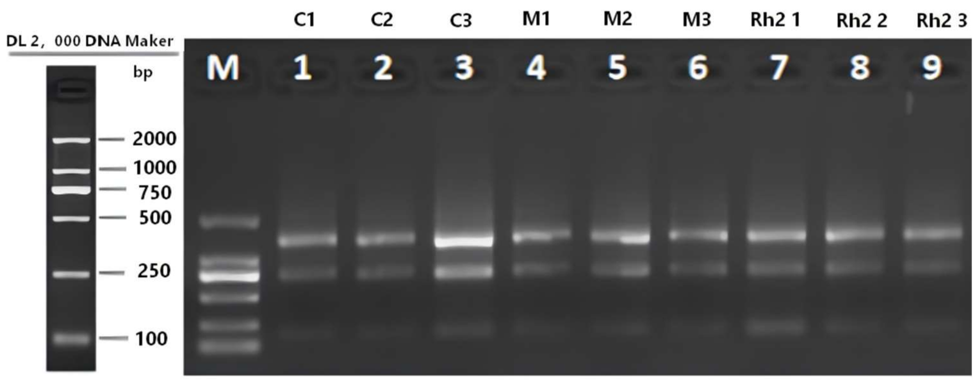

Supplementary Fig S3: Agarose gel electrophoresis analysis of mouse lung tissue.

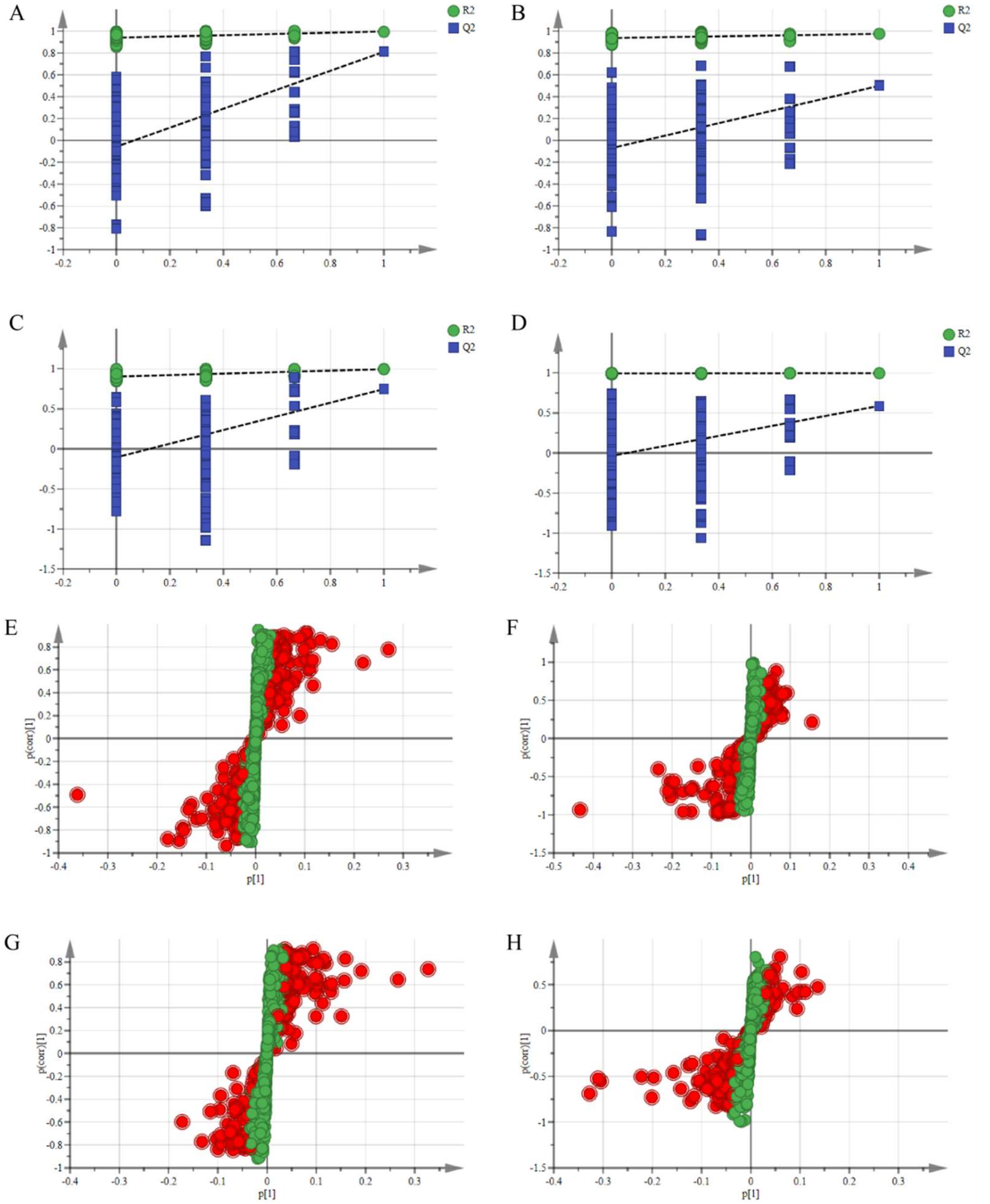

Supplementary Fig S4: Permutation test and S-plot for each group

permutation test plot in positive ion mode C vs. M (**A**), with permutation regression line R^2 = 0.94, Q^2 = -0.0565; permutation test plot in positive ion mode M vs. Rh_2 (**B**), with permutation regression line R^2 = 0.937, Q^2 = -0.0691; permutation test plot in negative ion mode C vs. M (**C**), with permutation regression line R^2 = 0.902, Q^2 = -0.105; permutation test plot in negative ion mode M vs. Rh_2 (**D**), with permutation regression line R^2 = 0.994, Q^2 = -0.0352; S-plot of C vs. M in positive ion mode (**E**); S-plot of C vs. M in positive ion mode (**F**); S-plot of C vs. M in negative ion mode (**G**); S-plot of C vs. M in positive ion mode (**H**).

Supplementary Table S1 Behavioral Scoring Scale

Manifestations of Asthma Attack in Mice	score
No asthmatic behavioral manifestations with shiny fur	0
Cyanosis of lips, tachypnea, occasional nose-scratching, normal coat	1
Labial cyanosis, tachypnea, restlessness, frequent nose scratching, slightly ruffled fur	2
Poor and ruffled coat, weight loss, sluggish response, severe dyspnea, persistent nose scratching	3

Supplementary Table S2 Behavioral scores of each group

Group	C	M	Rh2L	Rh2M	Rh2H	DXM
Score	0	2.67±0.52	2.17±0.41	1.33±0.52	1.17±0.41	1.17±0.41

Supplementary Table S3 Measurement of RNA concentration

Group	C1	C2	C3	M1	M2	M3	Rh2 1	Rh2 2	Rh2 3
TIC(ng/mL)	54.6395	82.3019	66.7462	2081.2554	684.3699	90.7551	2163.0142	458.0200	3190.6003
